# Divergences in gene repertoire among the reference *Prevotella* genomes derived from distinct body sites of human

**DOI:** 10.1186/s12864-015-1350-6

**Published:** 2015-03-05

**Authors:** Vinod Kumar Gupta, Narendrakumar M Chaudhari, Suchismitha Iskepalli, Chitra Dutta

**Affiliations:** Structural Biology & Bioinformatics Division, CSIR- Indian Institute of Chemical Biology, 4, Raja S. C. Mullick Road, Kolkata, 700032 India; Department of Pharmacoinformatics, National Institute of Pharmaceutical Education and Research, 4, Raja S. C. Mullick Road, Kolkata, 700032 India

**Keywords:** *Prevotella*, Pan-genome, Human microbiome

## Abstract

**Background:**

The community composition of the human microbiome is known to vary at distinct anatomical niches. But little is known about the nature of variations, if any, at the genome/sub-genome levels of a specific microbial community across different niches. The present report aims to explore, as a case study, the variations in gene repertoire of 28 *Prevotella* reference genomes derived from different body-sites of human, as reported earlier by the Human Microbiome Consortium.

**Results:**

The pan-genome for *Prevotella* remains “open”. On an average, 17% of predicted protein-coding genes of any particular *Prevotella* genome represent the conserved core genes, while the remaining 83% contribute to the flexible and singletons. The study reveals exclusive presence of 11798, 3673, 3348 and 934 gene families and exclusive absence of 17, 221, 115 and 645 gene families in *Prevotella* genomes derived from human oral cavity, gastro-intestinal tracts (GIT), urogenital tract (UGT) and skin, respectively. Distribution of various functional COG categories differs significantly among the habitat-specific genes. No niche-specific variations could be observed in distribution of KEGG pathways.

**Conclusions:**

*Prevotella* genomes derived from different body sites differ appreciably in gene repertoire, suggesting that these microbiome components might have developed distinct genetic strategies for niche adaptation within the host. Each individual microbe might also have a component of its own genetic machinery for host adaptation, as appeared from the huge number of singletons.

**Electronic supplementary material:**

The online version of this article (doi:10.1186/s12864-015-1350-6) contains supplementary material, which is available to authorized users.

## Background

The genetic script of any microorganism normally portrays a complex interplay between its taxonomic legacy and ecological prerequisites. The legacy of the ancestral gene repertoire should not vary within a specific lineage, but adaptation to distinct ecological niches often causes wide differentiation among closely related genomes through selection of conspicuous genetic traits. Microbes under adaptive evolution often undergo a process of genomic homeostasis - some old ancestral genes are shed off and new genes are acquired through lateral transfer [1-4]. There may also occur other evolutionary processes like recombination, gene duplication, and/or positive selection in specific genes, which, together with neutral mutation and drift; may bring about substantial genomic diversity between two species of the same genus, or even between two strains of the same species [5-10].

In a human body, the distinct body sites create unique niches for the resident microbiota that experience selective evolutionary pressures from the host as well as from other microbial competitors [11]. The nature of this pressure is likely to vary at different habitats, since the host cell environment and the microbiome's taxonomic composition both differ drastically from one body site to another. It is well known that local environmental filtering can have a great impact on *in situ* evolution of the microbial flora at distinct body niches [12]. In recent years, there has been an increasing amount of literature on adaptive evolution of the microbiome at different human body habitats, especially at the gastrointestinal tracts [12-15].

However, most of these studies have focused on the habitat-specific variations in the microbiome composition at the phylum, genus or species levels only and not much information is available on the variations, if any, at the genome/sub-genome levels of the resident microbes, though there are reasons to believe that adaptive strategies of these microbes at distinct niches might have been genomically encoded [13]. Release of an initial catalog of 178 initial reference genome sequences from the microbial flora of diverse anatomical niches in 2010 provided an opportunity of probing at niche-specific variations, if any, in the genome architectures of the human microbiota. Here, as a case study, we report the pan-genomic analysis of twenty-eight *Prevotella* genomes derived from different body sites of human and reported in this catalog [16]. The primary objective of the study was to explore the habitat-driven changes in the gene complements of these 28 *Prevotella* genomes.

*Prevotella* is a genus of Gram negative bacteria. It’s mainly composed of obligatory anaerobic bacilli. Based on biochemical differences in phenotypic characteristics like saccharolytic potential and bile sensitivity, and 16S rRNA gene phylogeny some species from *Bacteroides* were reclassified into a new taxonomical genus *Prevotella* [17]. The rationale behind selection of the genus of *Prevotella* as the case study lies in its importance as a component of the natural human flora. A study by Wu et al. [18] showed a strong association between the relative occurrences of the gut enterotypes with long-term diets of their respective hosts - the *Bacteroides* and *Prevotella* enterotypes being associated with protein and animal fat or carbohydrates, respectively. De Fillipo et al. reported exclusive presence of *Prevotella* and two other genera in rural African children having fiber rich diets while *Bacteroides* were absent [19]. Changes in *Prevotella* abundance and diversity may also occur during several dysbiosis-associated diseases, including bacterial vaginosis, asthma and chronic obstructive pulmonary disease (COPD) and rheumatoid arthritis [20-22]. *Prevotella* spp. are often implicated in diverse anaerobic infections arising from the respiratory tract, urogenital tract and gastrointestinal tract [23,24].

Significance of *Prevotella* as a component of human microbiota is, therefore, beyond doubt. Yet, little is known about the genetic basis of *Prevotella* diversity at different body habitats of humans and its symbiotic/pathogenic implications. To this end, we have made a pan-genome analysis of 28 *Prevotella* genomes derived from distinct body habitats like oral cavity, gastrointestinal tract (GIT), urogenital tract (UGT) and skin. The concept of the pan-genome analysis [25,26], though traditionally applied to delineate the complete repertoire of genes in different strains of a single species [27,28] has recently been extended to represent the total gene complements in any pre-defined group of microorganisms [29,30]. In the present endeavor, an attempt has been made to delineate the core gene complements as well as the habitat specific variations in accessory or dispensable genome composition, if any, in 28 *Prevotella* genomes derived from distinct body habitats like oral cavity, gastrointestinal tract (GIT), urogenital tract (UGT) and skin. The analysis has revealed not only the habitat-specific presence, but also habitat-specific absence of certain gene families in *Prevotella*. Distinct trends have also been observed in distribution of various functional clusters of orthologous groups (COGs) between the core, flexible and unique genes within the *Prevotella* genomes as well as between the GIT derived *Bacteroides* and *Prevotella*. It appears that distinct selection pressures, as imposed by the specific niches within the host body, play an important role in shaping the genetic make-up of individual microbiome members.

## Results

### Orthologous gene families - classification into the core, flexible, and singleton genes

The dataset of “*Prevotella* Draft Genomes from Human Microbiome” (PDGHM), used in the present analysis, contains 28 annotated genome assemblies from 25 *Prevotella* species, isolated from oral cavity, GIT, UGT and skin microbiome of human (Table 1).

**Table 1:** **Details of** ***Prevotella*** **strains used for this analysis**

**Sr. No.**	**Name of organism**	**Niche**	**BioProject accession**	**SL**	**Size (Mb)**	**GC (%)**	**CDS Count**	**% Core genes**	**% Acc. genes**	**% Unique genes**	**N50 (Kb)**	**% Bacterial core genes out of 200**
1	*P. copri DSM 18205*	GIT	PRJNA30025	S	3.51	46.3	3337	14	41	45	442.3	98
2	*P. salivae DSM 15606*	GIT	PRJNA53199	C	3.27	42.4	2939	16	62	22	3180.2	94.5
3	*P. stercorea DSM 18206*	GIT	PRJNA65131	S	3.10	50.5	3017	15	39	46	53.8	96.5
4	*P. buccae ATCC 33574*	Oral	PRJNA51491	S	3.28	52.2	2896	16	66	18	3266.2	94.5
5	*P. buccae D17*	Oral	PRJNA38737	S	3.36	52.4	2617	17	73	10	151.3	92.5
6	*P. denticola F0289*	Oral	PRJNA49293	F	2.94	51.9	2386	19	73	8	2937.6	94.5
7	*P. marshii DSM 16973*	Oral	PRJNA50531	S	2.56	48.5	2335	20	52	28	2544.6	98
8	*P. melaninogenica ATCC 25845*	Oral	PRJNA31383*	F	3.17	42.8	2296	20	73	7	1796.4	94.5
9	*P. melaninogenica D18*	Oral	PRJNA40045	S	3.29	42.7	2461	19	72	9	217.1	94.5
10	*P. multiformis DSM 16608*	Oral	PRJNA53055	S	3.06	52.9	2884	16	61	23	1620.3	94.5
11	*P. nigrescens ATCC 33563*	Oral	PRJNA64737	S	2.67	44	2421	19	63	18	2084	96.5
12	*P. oris F0302*	Oral	PRJNA38329	S	3.25	45	3316	14	61	25	432.3	96.5
13	*P. oulorum F0390*	Oral	PRJNA43117	S	2.81	47.8	2488	18	57	25	1512.2	98
14	*P. pallens ATCC 700821*	Oral	PRJNA64739	S	3.13	38.8	2860	16	61	23	2314.9	94.5
15	*P. sp. oral taxon 299 str. F0039*	Oral	PRJNA40047	S	2.45	38.7	1935	24	49	27	580.3	96.5
16	*P. sp. oral taxon 306 str. F0472*	Oral	PRJNA75153	S	2.95	42.7	2442	19	72	9	127.9	93.5
17	*P. sp. oral taxon 317 str. F0108*	Oral	PRJNA38521	S	4.10	49.1	2926	16	64	20	783.3	94.5
18	*P. sp. oral taxon 472 str. F0295*	Oral	PRJNA38731	S	3.64	48.2	3092	15	58	27	1184.5	96.5
19	*P. tannerae ATCC 51259*	Oral	PRJNA33153	S	2.59	47.5	2811	16	27	57	1429.3	94.5
20	*P. veroralis F0319*	Oral	PRJNA38331	S	2.99	43	3048	15	63	22	187	94.5
21	*P. bergensis DSM 17361*	Skin	PRJNA34637	S	3.27	48.9	2825	16	51	33	1075.7	94.5
22	*P. amnii CRIS 21A-A*	UGT	PRJNA40671	C	2.42	37.7	2023	23	62	15	88.5	95.5
23	*P. bivia JCVIHMP010*	UGT	PRJNA31377	C	2.42	41.1	2041	22	63	15	48.6	96
24	*P. buccalis ATCC 35310*	UGT	PRJNA40669	C	3.03	46.8	2456	19	61	20	74.5	95.5
25	*P. denticola CRIS 18C-A*	UGT	PRJNA61825	C	3.18	51.4	2701	17	74	9	97.1	94.5
26	*P. disiens FB035-09AN*	UGT	PRJNA51065	C	3.00	41.5	2621	17	63	20	128	96.5
27	*P. oralis ATCC 33269*	UGT	PRJNA51495	S	2.84	45.6	2488	18	53	29	2169.4	94.5
28	*P. timonensis CRIS 5C-B1*	UGT	PRJNA40667	C	2.80	43.6	2202	21	61	18	25.9	94.5

GIT- Gastrointestinal Tract, UGT- Urogenital Tract, *- Two Chromosomes, C- Contigs, S- Scaffolds, F- Finished, SL- Sequencing Level, % Acc Genes- % Accessory Genes.

Three species namely *P. buccae, P. denticola* and *P. melaninogenica,* have the genomes of two strains each, while the other 22 species have only one strain each in this dataset. Total genome sizes and the number of predicted protein coding sequences in PDGHM vary between species and ranged from 2.42 to 4.1 Mb and 1935 to 3337, respectively (Table 1). Interestingly, the genome sizes and the number of CDSs of seven *Prevotella* genomes derived from the urogenital tracts (UGT) are in general lower than those of three gut isolates, while the genomes isolated from the oral cavity vary widely in the genome sizes as well as in the number of predicted CDSs. The average genomic G + C content also varies widely across the PDGHM dataset – from 38.7% in *Prevotella sp. oral taxon 299 str. F0039* to 52.2% in another oral derivative *P. buccae ATCC 33574*. There are substantial intra-species variations in genome sizes and number of CDSs also across two strains of *P. buccae, P. denticola* and *P. melaninogenica* (Table 1). Existence of significant variations in G + C-content, genome size and number of CDSs is not surprising in view of the fact that the genus *Prevotella* is yet to have a robust taxonomic outline and it is in need of revision [31].

A total of 73864 annotated complete CDSs of PDGHM, when clustered by the CD-HIT algorithm [32] yielded 24885 distinct clusters of orthologous genes (gene families). Members of these gene families have been categorized into two sets based on their occurrences in different *Prevotella* genomes under study: (i) the “core” genes that exist in all 28 members of PDGHM and (ii) the “dispensable” or “flexible” genes that exist in some, but not in all *Prevotella* genomes under study. The dispensable genes may again be classified into two categories: (a) the “singleton” or “unique” genes, which are specific to single genomes, and (b) the “accessory” genes that are present in more than one, but not in all genomes of PDGHM. Among 24885 gene families, 456 families (~1.81%) exist in all twenty-eight genomes and hence represent the core gene complements of the PDGHM dataset (Additional file 1: Figure S1). The number of predicted protein-coding genes in individual *Prevotella* genomes lies within 2638 ± 700, and on an average, 17% of these genes represent the conserved core genes. 7263 gene families (~29% of the pan genome) comprise the set of accessory genes, found in more than one, but not in all *Prevotella* genomes of the current dataset (Additional file 1: Figure S1).

A huge percentage (~69%, 17166 gene families) of the total gene repertoire in the pan-genome are present in only one genome (Additional file 1: Figure S1). Among these, only 4972 are functionally annotated and 12194 are hypothetical gene families. The number of these unique genes or singletons varies significantly across different *Prevotella* genomes of PDGHM (Table 1). In the oral cavity isolate *P. tannerae*, 57% of the annotated CDS appeared to be singletons with no orthologs in other PDGHM members, as per the 50% similarity & 50% coverage criteria. Two GIT isolates *P. copri* and *P. stercorea* also have shown substantially high percentage of predicted CDSs in the unique gene category, while in the sole skin isolate *P. bergensis*, 33% of the CDSs have been identified as unique genes. The percentage of unique genes is significantly low in *P. buccae, P. denticola* and *P. melaninogenica*, as these species share the species-specific genes between their two strains. Numbers of unique genes are also relatively low (<20%) in all UGT isolates except *P. oralis ATCC 33269* (Table 1). A complete list of strain wise core, accessory and unique genes is shown in the Additional file 2: Table S1.

### Pan genome and core genome plots

**Figure 1:**
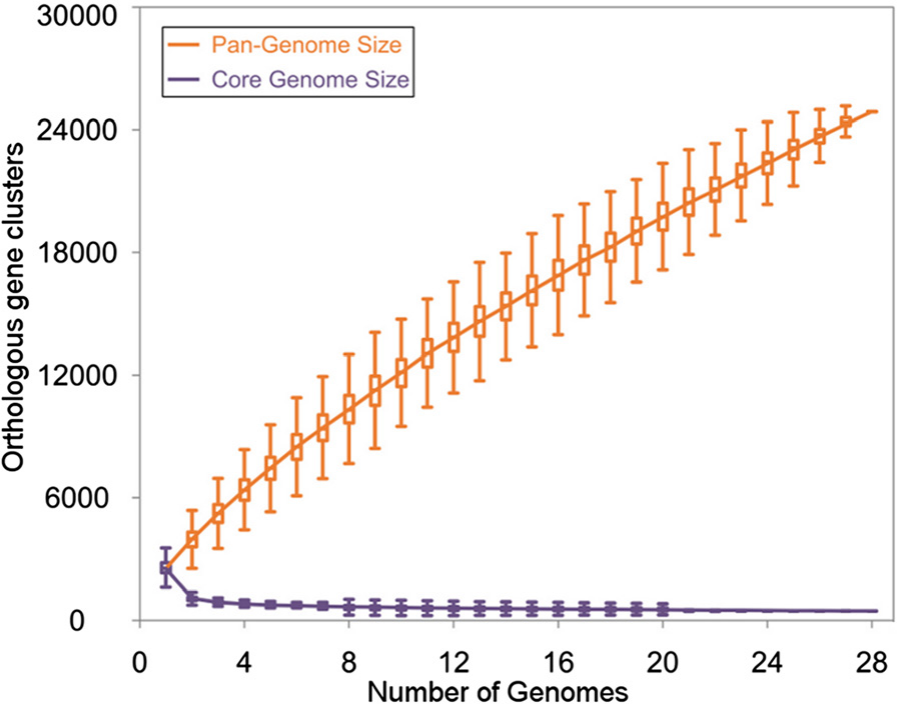
**Pan and core genome analysis of 28** ***Prevotella*** **genomes.** The number of shared genes is plotted (violet) as a function of the number of *Prevotella* genomes sequentially considered. The continuous curve represents the calculated core genome size, exponential curve fit model (*y*_*core*_ *= A*_*core*_ *e* ^*Bcore.x*^ *+ C*_*core*_) was applied to the data. The best fit was obtained with *r* ^2^ = 0.949, A_core_ = 5490.32, B_core_ = −1.05, and C_core_ = 567.29. The extrapolated *Prevotella* core genome size is 567. The size of *Prevotella* pan-genome is plotted (orange) as a function of the number of *Prevotella* genomes sequentially considered. The continuous curve represents calculated pan-genome size, the power-law regression model (*y*_*pan*_ *= A*_*pan*_ *x* ^*Bpan*^ *+ C*_*pan*_) was applied to the data. The best fit was obtained with *r* ^2^ = 0.999, A_pan_ = 2389.18, B_pan_ = 0.7, and C_pan_ = 66.29. The extrapolated *Prevotella* pan-genome size is 24685. The vertical bars correspond to standard deviations after repeating random combinations of the genomes.

With a view to study the expansion of the pan-genome of PDGHM with sequential addition of more *Prevotella* genomes in the dataset, we have plotted the total number of distinct gene families against the number of genomes considered (Figure 1). Similarly, the number of shared gene families has been plotted against the number of genomes in order to generate the core-genome plot that depicts the trend in contraction in the core genome size with sequential addition of more genomes. In order to avoid any bias in the sequential addition of new genomes, random permutations in the order of addition of genomes were carried out and a median was taken on the size of pan-genomes or core genomes after each step (Figure 1). The median counts were then extrapolated using the power-law regression model for pan-genome and an exponential curve fit model in case of the core genome (see Methods for details). As depicted in Figure 1, the size of the pan-genome increases unboundedly with addition of new genomes and even after inclusion of 24885 non-redundant gene-families from all 28 members of PDGHM, the plot is yet to reach a plateau. On an average, each additional PDGHM genome contributed 827 new genes to the pool, leading to an open pan-genome. In accordance with these observations, the power-law regression shows that the pan-genome of PDGHM is indeed “open” [33] with a γ-parameter of 0.7 (here, *B*_*pan*_).

As expected, the size of core genome gradually decreases with inclusion of each new genome and the curve, though gradually approaching a plateau, has not reached it fully (Figure 1). This indicates that the core genome of PDGHM is yet to arrive at a “closed” state, i.e., addition of new *Prevotella* genomes to PDGHM may result in further contraction in its core genome.

As shown in Table 1, the PDGHM dataset contains 17 *Prevotella* genomes isolated from the oral cavity, 7 from urogenital tract, 3 from gut and 1 from skin of the human. Our next objective was to examine whether the trends in expansion of the pan-genome and/or contraction of the core genome differ across human body niches. To this end, we generated the pan genome and core genome plots once again. But this time no permutations of the genomes was carried out, as that would prevent visualization of the progression across niche-specific subsets. Instead, genomes isolated from a specific body niche were added serially (Additional file 3: Figure S2a). The plot started with GIT isolates and then gradually the isolates from oral cavity, skin and UGT were added. As can be seen in Additional file 3: Figure S2a, the GIT-derived genomes added an appreciable number of new genes to the pan-genome. But subsequent addition of the *Prevotella* genomes from different body-sites did not cause any drastic change in the trends in expansion of the pan-genome or reduction of the core genome, except in the case of the oral isolate *P. tannerae,* inclusion of which led to a sharp decrease in the core gene number and also to the addition of an appreciable number of new genes to the pan-genome (Additional file 3: Figure S2a). These observations are in good agreement with the findings that *P. tannerae* contains the highest percentage of unique genes; followed by two GIT isolates *P. copri* and *P. stercorea* (Table 1).

In Additional file 3: Figure S2a, the shape of the pan and core genome curves would be different for a different ordering of the genomes. In order to ensure that the trends observed in Additional file 3: Figure S2a were not artifacts, we tried both inter-niche and intra-niche variations in the ordering of genomes in core and pan genome plots (Additional file 3: Figure S2b). In all cases, the trends inferred from Additional file 3: Figure S2a remained valid. For instance, in all cases, the size of the pan genome increased substantially with inclusion of GIT isolates. Appreciable increase in the pan genome size and decrease in the core gene numbers upon addition of *P. tannerae* is also apparent in all permutations in genome ordering (Additional file 3: Figure S2b). The end-points of all the core and pan genome plots were also same as in Figure 1, keeping the estimates of the size of the core and pan-genomes unaltered.

### Exclusive presence or absence of gene families in genomes derived from specific body sites of human hosts

**Figure 2:**
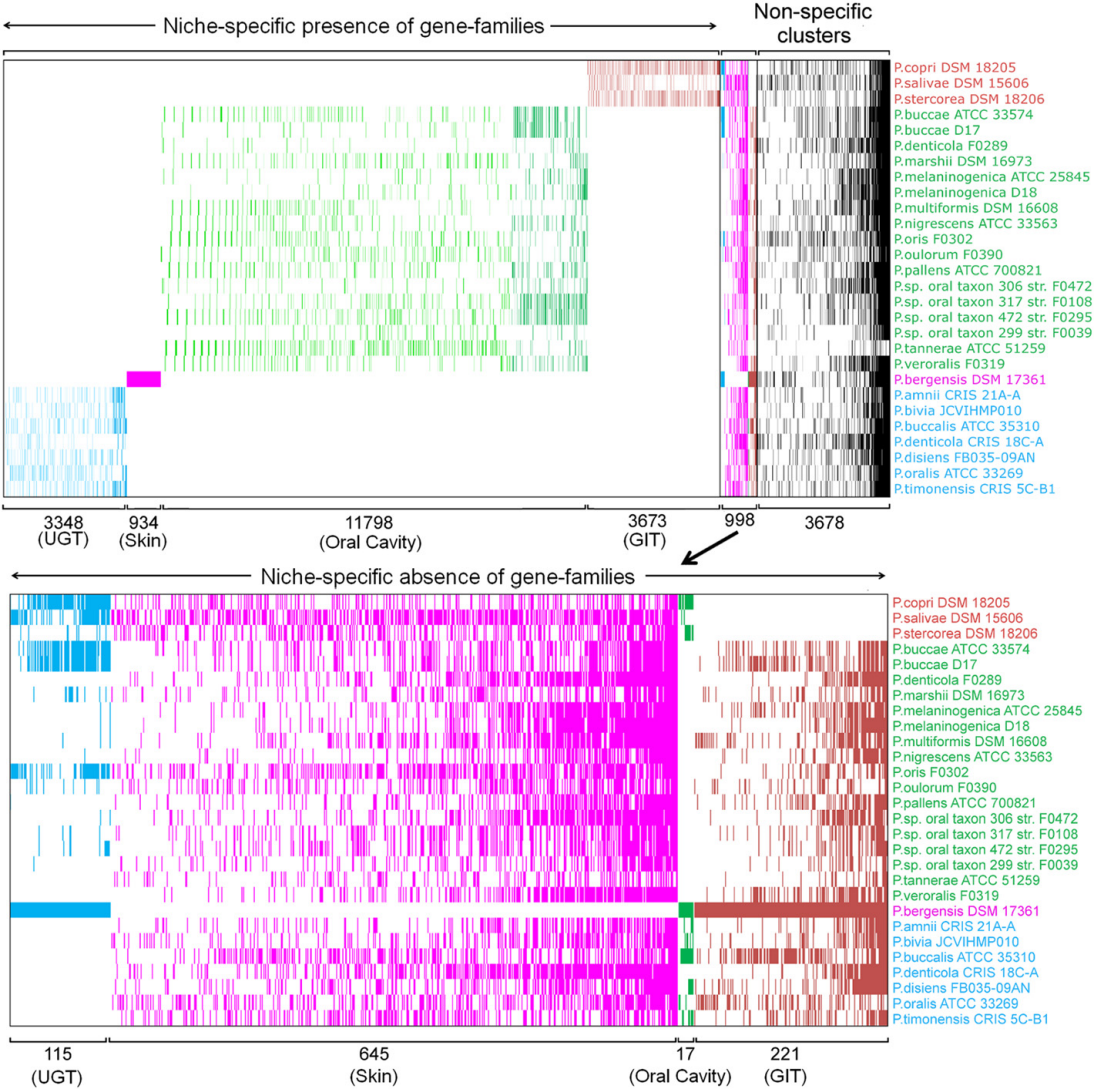
**Distribution of dispensable genome among 28** ***Prevotella*** **strains.** Colored cells indicate presence of genes in the respective *Prevotella* strain and orthologous gene family, while uncolored cells indicate absence of genes. The species names and cells are colored according to their niches - brown: GIT, green: ORAL Cavity, purple: SKIN and blue: UGT. Dark cell colors represent flexible genome and light cell colors represent singletons.

To investigate the genomic and proteomic diversity between *Prevotella* species adapted at different body sites of human, we have constructed the binary gene presence/absence matrices for orthologous gene families within these smaller niche-specific datasets. Interestingly enough, there are 19753 families showing habitat-specific presence, i.e., exclusive existence in the genomes isolated from a specific site of the human body (niche specific clusters). As depicted in Figure 2, there are 11798, 3673, 3348 and 934 gene clusters, which have members exclusively present in the *Prevotella* genomes derived from human oral cavity, GIT, UGT and skin, respectively (Table 2, Figure 2). It was not surprising to find largest number of habitat-specific gene families (11798) in oral isolates, since 17 genomes out of 28 in our study belonged to the oral cavity. A complete list of these niche-specific genes is shown in the (Additional file 4: Table S2).

**Table 2:** **Niche specific** ***Prevotella*** **pan-genome**

**Niche**	**No. of genomes**	**Orthologous clusters (Pan genome size)**	**Core clusters**	**Niche specific clusters**	**Exclusively absent clusters**
GIT	3	6431	927	3673	221
ORAL	17	16461	513	11798	17
SKIN	1	-	-	934	645
UGT	7	7203	808	3348	115

GIT: Gastrointestinal Tract, Oral: Oral Cavity, UGT: Urogenital tract.

It was even more intriguing to find 998 gene families that are absent exclusively in genomes derived from specific body sites of the host (Figure 2). As shown in the zoomed portion of Figure 2, there are 221 gene families, which are present in all PDGHM members derived from oral cavity, skin and UGT, but not in those derived from GIT. Similarly, there are 17, 115 and 645 genes specifically absent in oral, UGT and skin isolates of PDGHM. This observation suggests that adaptation to any specific niche within the host body might require both the gain and loss of specific genes in the microbiota. A list of the genes that are absent from genomes derived from specific body habitats of humans is provided in the (Additional file 5: Table S3).

We have also identified the gene families shared by all members of different subsets of PDGHM isolated from specific host body sites. Numbers of such core gene families in GIT, oral cavity and UGT derivatives were 927, 513 and 808, respectively. Number of total gene families (i.e. pan genomes) in GIT, oral cavity and UGT subsets of PDGHM were 6431, 16461 and 7203 respectively. A complete list of genes absent from each *Prevotella* strain is shown in Additional file 6: Table S4.

### Trees based on the pan - matrix and core genome – niche-specific features

**Figure 3:**
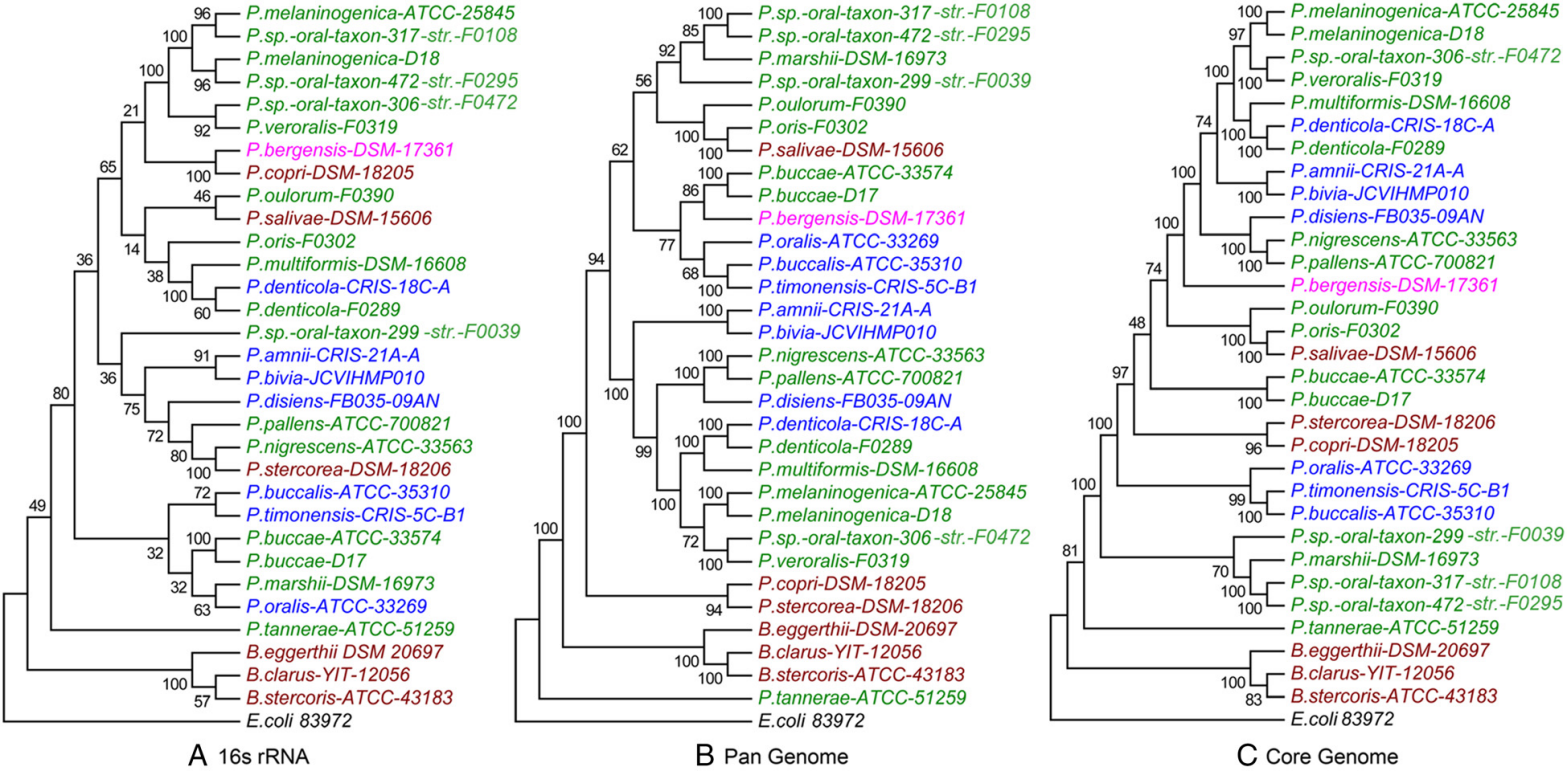
**Relative evolutionary divergence of** ***Prevotella*** **. (A)** Neighbor Joining (NJ) tree based on 28 *Prevotella* and *E. Coli 83972* (reference) 16S rRNA sequences, was constructed using MEGA 6 after 1000 bootstrap replications, **(B)** NJ Tree based on the binary gene presence/absence matrix of orthologous gene families of 28 *Prevotella* and 3 *Bacteroides* strains and **(C)** NJ tree based on core genome using 100 bootstrap replications. The bootstrap values are marked at the root of each branch of trees. The species names are colored according to their niches (brown: GIT, green: ORAL Cavity, purple: SKIN and blue: UGT).

In an attempt to elucidate the relative importance of lineage-specific divergences and niche-specific selections in shaping the gene architectures of the PDGHM, we have constructed three Neighbor Joining (NJ) Trees (Figure 3). The first one is the traditional phylogenetic tree generated from 16S rRNA sequences (Figure 3A), the second tree is based on the binary gene presence/absence matrix (Figure 3B) and the third one has been constructed using concatenated alignments of core genes (Figure 3C). In all three trees, *E. coli* has been taken as the outgroup species and 3 GIT-derived *Bacteroides* genomes have been included in an attempt to see whether the GIT-derived genomes of *Prevotella* and *Bacteroides* cluster together. These 3 *Bacteroides* genomes are selected as they are similar in genomic properties like GC content and genome size to that of GIT derived *Prevotella* isolates. *Bacteroides* from other body sites are not included in our analysis due to unavailability of complete genome sequences. Sequences isolated from the gut, oral cavity, skin and urogenital tract are highlighted in brown, green, purple and blue color respectively.

In all three trees (Figure 3), three *Bacteroides* members segregated together under a distinct node – completely separated from the *Prevotella* genomes. This observation suggests that so far the genetic architectures of the microbiome components are concerned, the taxonomic legacy rules over their niche-based needs, if any. Though in figure 3, for sake of resolution we have included only three GIT-derived *Bacteroides* genomes as representative examples, it has been checked that lineage-specific segregation of *Bacteroides* from *Prevotella* does not depend on choice of representative *Bacteroides* genomes. Conspicuous standing out of *P. tannerae*, either next to or in between *E. coli* and *Bacteroides* in all three trees is quite consistent with the recent reassignment of *P. tannerae* under a novel genus *Alloprevotella* gen. nov [34].

Interestingly enough, the trends of segregation of *Prevotella* genomes into different sub-groups in the pan-matrix based tree (Figure 3B) and the Core genome based tree (Figure 3C) bear a high resemblance, though the relative positions of the sub-groups differ substantially in two trees. A comparison of these two trees with 16S rRNA tree (Figure 3A) revealed a number of similarities as well as divergences. In all three trees, the oral isolates *P. oulorum* and *P. oris* and the GIT isolate *P. salivae* appeared either under a common node (Figure 3B and C), or adjacent to each other (Figure 3A). Two UGT isolates, *P. amnii* and *P. bivia* segregated under a common node. Two other UGT isolates, *P. buccalis* and *P. timonensis* also co-segregated in all trees.

These observations pointed out that the gene repertoire of the individual genomes as well as the core genome in these microbiome components are in good agreement with their 16S rRNA phylogeny.

Comparison of three trees also reveals a number of niche-specific divergences. *P. copri DSM 18205* and *P. stercorea DSM 17361*, despite substantial distances in 16S rRNA tree, co-segregated in the pan-matrix based tree and core genome based trees, suggesting that gene content of these two “not-so-closely-related” GIT isolates might have played some role in their adaptation to a similar habitat within the host body. Intriguingly enough, these two GIT-derived *Prevotella* genomes appeared in a node adjacent to the node of three GIT-derived *Bacteroides* in the pan-matrix based tree (Figure 3B), which suggests that there might be certain inter-genus similarities in the gene repertoire of these GIT-derived bacteria. However, as mentioned above, such habitat-specific similarities could not hinder lineage-specific segregation of *Prevotella* and *Bacteroides* genomes.

We have also studied the trends in codon usage in the core gene sets for each of the *Prevotella* genomes under study and calculated the codon usage distances between the core gene sets for all possible pairs of genomes, as described in Methods. A heat-map of codon usage distances has been shown in the Additional file 7: Figure S3. It is clear from this heat-map that the codon usage in the core genes of *Prevotella* members merely reflects the average genomic G + C-bias of the respective genomes. Organisms having similar G + C-bias show lower values for codon usage distance, irrespective of their host body niches. In other words, adaptation to any specific anatomical niches of the human body might have influenced (or have been influenced by) the gene repertoire of the microbiome components under study, but it appears that such adaptation could not impart any niche-specific selection pressure at the codon levels, in general.

### Clustering of genomes on the basis of major KEGG pathways and COG distribution

**Figure 4:**
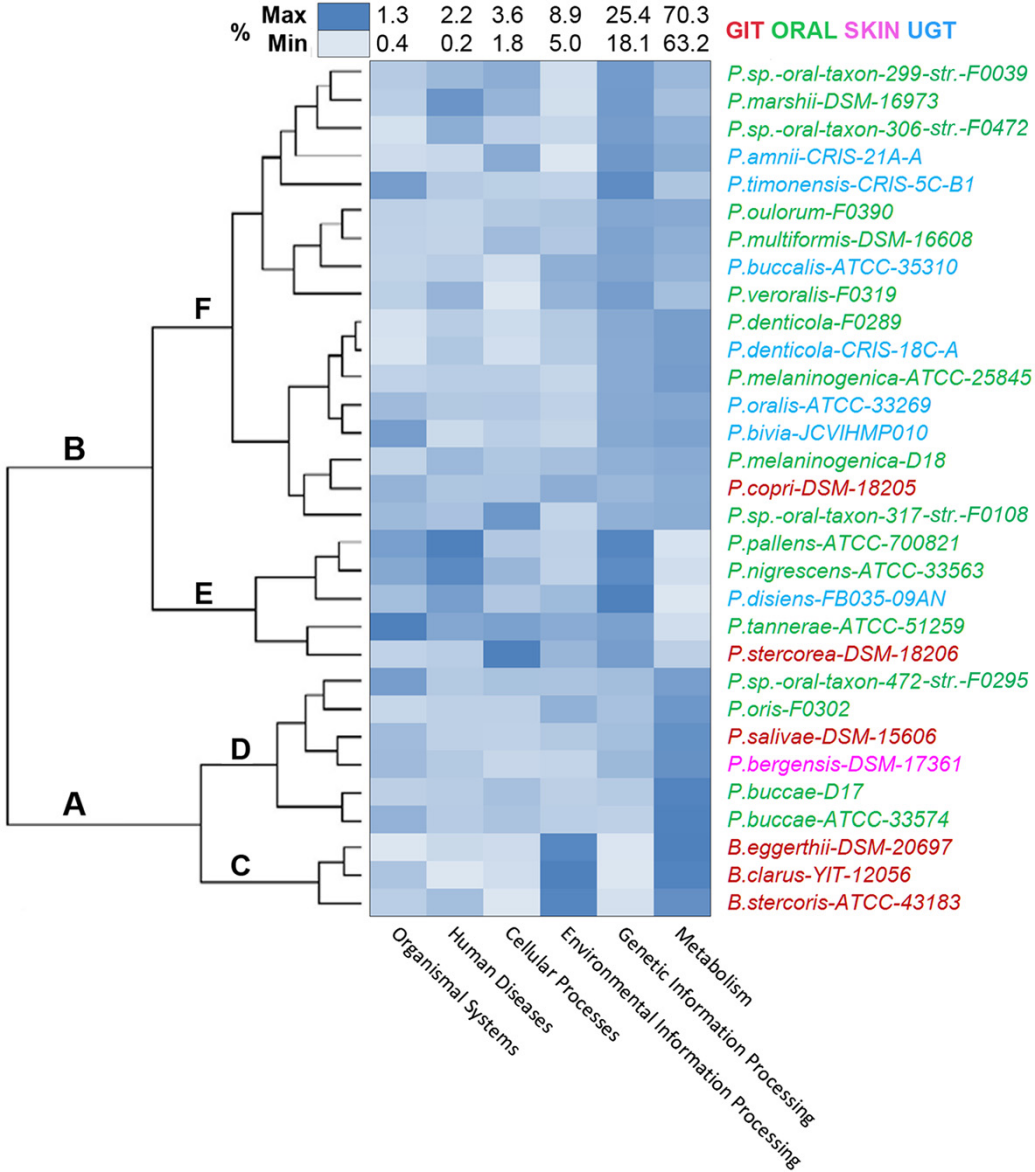
**KEGG pathway frequency heatmap.** All coding genes annotated against KEGG database and KEGG pathway frequencies were hierarchically clustered in two dimensions. The horizontal axis shows the percentage frequency of genes involved in respective pathways, while the strains are located on vertical axis.

**Figure 5:**
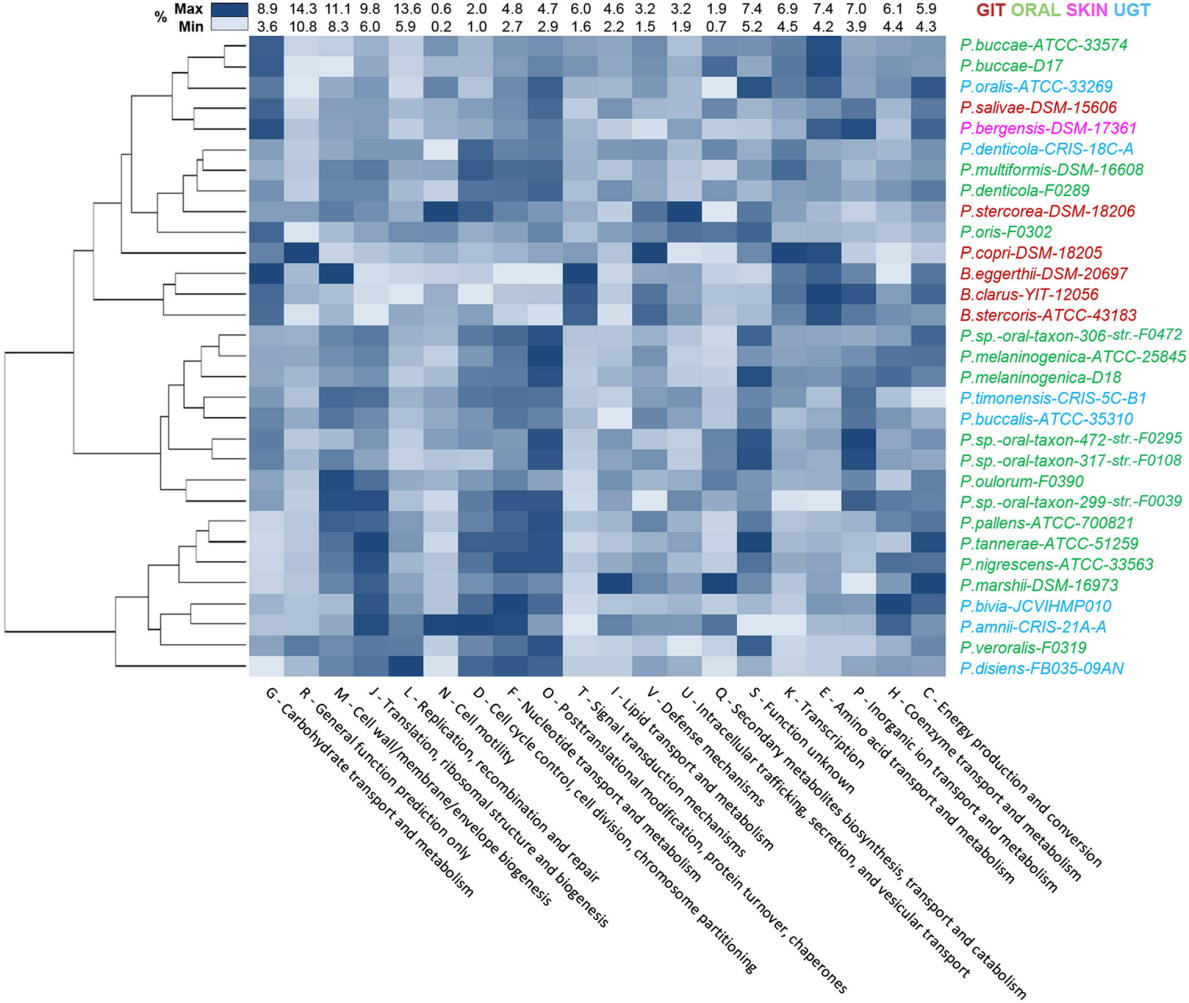
**COG frequency heatmap.** All coding genes annotated against COG database and frequencies of functional COG categories were hierarchically clustered in two dimensions. The horizontal axis shows the percentage frequency of genes involved in respective functional COG category, while the strains are located on vertical axis.

Realization of the fact that the lineage-specific selections and niche-specific constraints both might have played significant roles in shaping the genomic architectures of the microbiome components under study has prompted us to examine the distribution of KEGG pathways and major COG categories in 28 *Prevotella* genomes and 3 *Bacteroides* genomes of the current dataset. Clusters of 31 genomes generated on the basis of the distribution of major KEGG pathway categories and COG categories have been shown along with their heat-maps in Figure 4 and 5, respectively.

In Figure 4, the entire dataset has been bifurcated under two nodes. 3 *Bacteriodes* species, though segregated together under a distinct sub-node C, appeared along with 6 *Prevotella* genomes (four oral isolates, one skin and one GIT component) under one major node A, while all other *Prevotella* genomes have formed a separate cluster under the other node B. As shown in the heat-map, 3 *Bacteroides* components are characterized by higher occurrence of *Environmental information processing* pathways as compared to *Prevotella* genomes. All genomes under the node A, especially the *Bacteroides*, have relatively low frequencies of pathways pertaining to *Genetic information processing*, while the pathways involved in *Metabolism* appear to have low occurrences in genomes under the sub-node E. Genomes under the sub-node E (except *P. stercorea*) also show higher occurrences of *Human diseases* related pathways. There are other two *Prevotella* genomes, namely *P. marshii* and *P. sp. oral taxon 306* showing high frequencies of *Human diseases* related pathways.

Three *Bacteroides* representatives have segregated under a separate sub-node also in Figure 5, in which clustering has been carried out on the basis of relative occurrences of genes pertaining to different functional COG categories. As revealed in Figure 5, the *Bacteroides* are conspicuous for their high content of gene families involved in *Signal Transduction mechanisms* and relatively low contents of genes under the categories O and F (*Post translational modifications, protein turnover and chaperons* and *Nucleotide transport and metabolism*), as compared to the *Prevotella* organisms under study.

### Functional categories of the core genes, accessory genes, singletons and niche-specific genes

**Figure 6:**
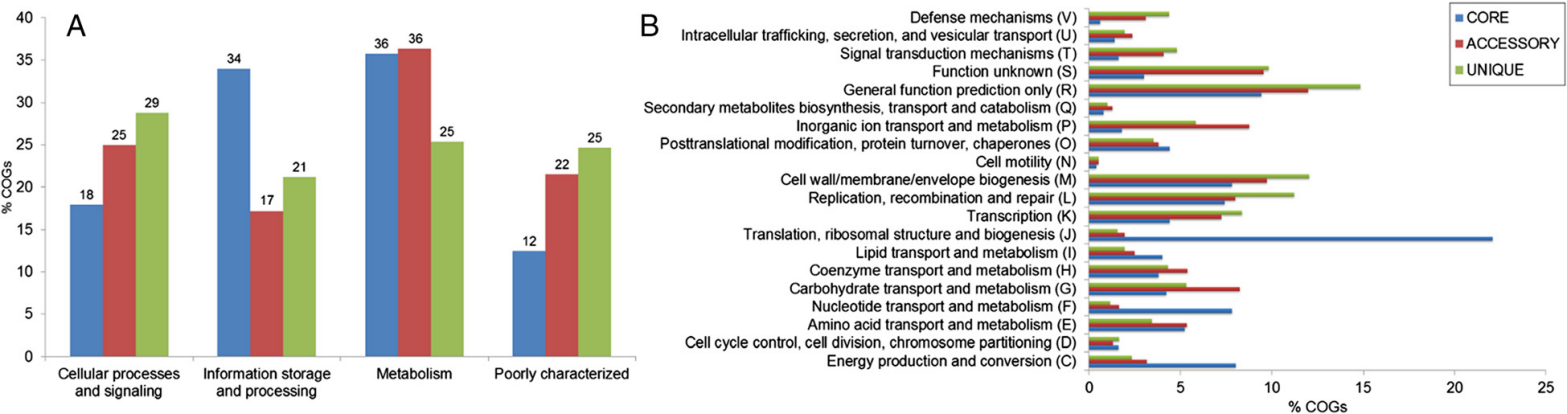
**Relative abundance and distribution of COG categories between core genome (blue bars), accessory genome (red bars) and singletons (green bars) of** ***Prevotella*** **. (A)** General COG categories, **(B)** Functional COG categories. Only orthologous gene families assigned by WebMGA server were used for analysis.

Our next objective was to assign core, accessory and unique genes of the PDGHM dataset to different functional categories, taking one representative sequence from each gene family. Figure 6A shows the distribution of the major COG categories in these three groups of genes. Majority of the core genes belong to the *Information storage and processing* (34%) and *Metabolism* (36%) categories*.* On the contrary, a major portion (29%) of the singletons belongs to the category of *Cellular processes and signaling*.

An examination to further details (Figure 6B) revealed that more than 22% of the core genes belong to the *Translation, ribosomal structure & biogenesis* (J) COG category. Members of the *Nucleotide transport & metabolism* (F) and *Energy production & conversion* (C) categories are also present in much higher percentage among the core genes (7.8% & 8%) than among the accessory (1.6% & 3.2%) or unique genes (1.2% & 2.3%). Disregarding the gene families under categories of *Unknown functions* (S) and *General function prediction only* (R), which include almost one-fourth of the singletons, majority of the singletons (31.6%) appear to be involved in *Cell wall/membrane/envelope biogenesis* (M), *Replication, recombination & repair* (L) and *Transcription* (K) processes (12%, 11.2%, & 8.4% respectively).

**Figure 7:**
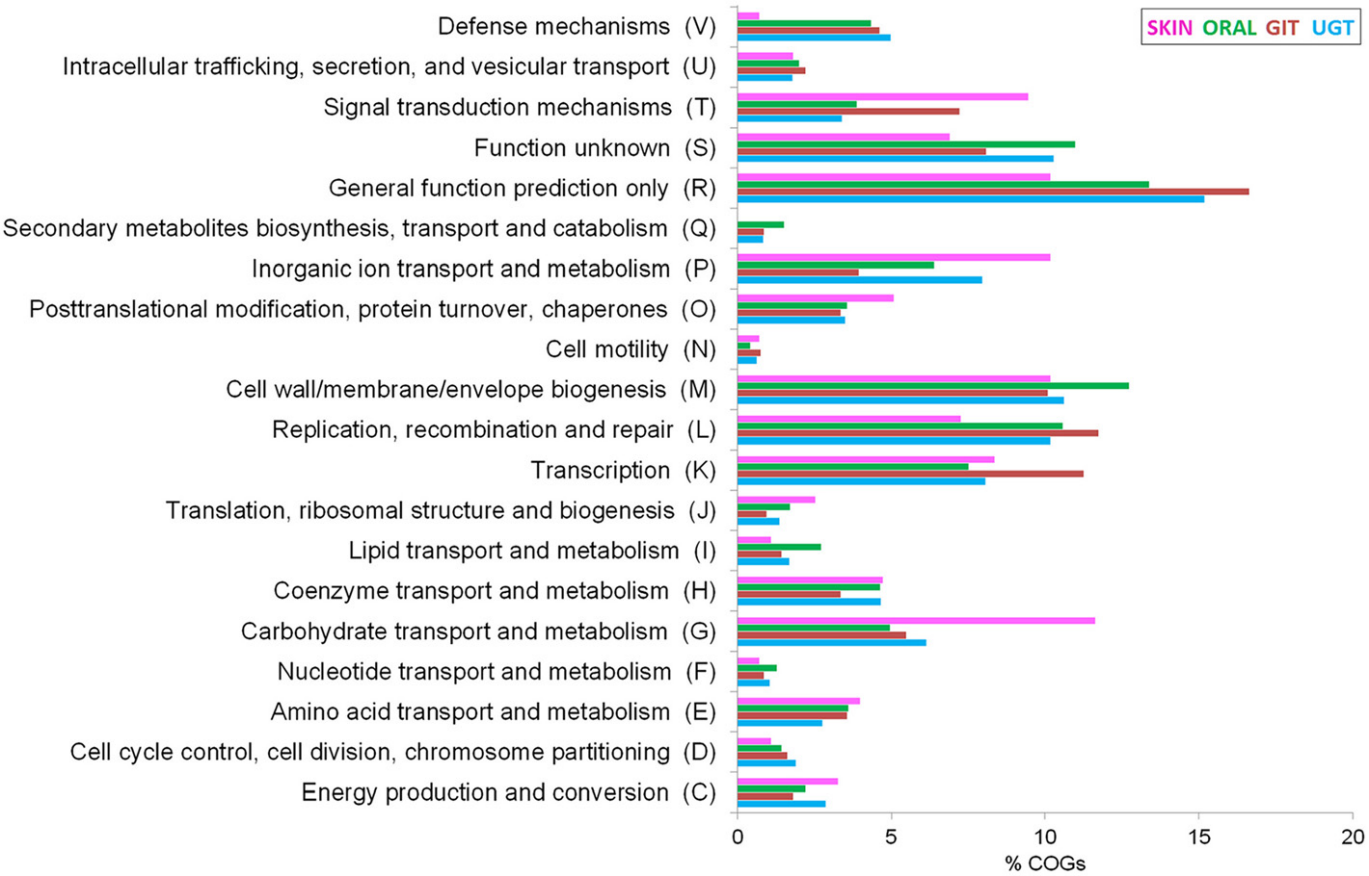
**COG distribution patterns of the niche-specific orthologous gene families.**

COG distribution patterns of the niche specific genes are presented in Figure 7. Certain COG categories like *Transcription* (K), *Replication, recombination and repair* (L), *Cell wall/ membrane/ envelope biogenesis* (M), are found in relatively higher frequencies (Figure 7), as compared to all other categories, among all habitats. Among the gene families found exclusively in the skin isolate, genes involved in *Signal transduction mechanisms* (T), *Carbohydrate transport and metabolism* (G) and *Inorganic ion transport and metabolism* (P) have significantly high frequencies (9.5%, p = 0.034, 11.6%, p = 0.007 & 10.2%, p = 0.016 respectively). GIT-specific gene families are significantly enriched in genes involved *Signal transduction mechanisms* (T, 7.5%, p = 0.106), *Replication, recombination and repair* (L, 12%, p = 0.007), *Cell wall/membrane/envelope biogenesis* (M, 10.3%, p = 0.016) and Transcription (K, 11.8%, p = 0.007), while genes under the category *Cell wall/membrane/envelop biogenesis* (M, 13.6%, p = 0.001) and *Replication, recombination and repair* are significantly (L, 11.6%, p = 0.007) more frequent among oral isolates (Figure 7). Interestingly enough, genes associated with *Defense mechanisms* (V, 0.7%, p = 0.005) are significantly underrepresented in the skin-specific families, as compared to those in exclusively present in GIT (4.4%, p = 0.178), oral cavity (4.4%, p = 0.178) and UGT isolates (5.5%, p = 0.180).

### Functional categorization of singletons of individual genomes

Functional categorization of the unique genes from individual genomes is further depicted in Additional file 8: Figure S4. COG distribution patterns of singletons varied widely across the genomes, showing no readily identifiable niche-specific features and in most of the genomes, a substantial fraction (~25%) of singletons fell under the categories of *General function prediction only* and *Function unknown* (R and S). Nevertheless, a careful analysis of the pie charts in Additional file 8: Figure S4 revealed certain intriguing trends. In majority of the *Prevotella* genomes including the sole skin derivative *P. bergensis* and three GIT isolates, a substantial fraction (~32%) of unique genes fall in the categories Transcription (K, 8.4%), *Replication, recombination and repair* (L, 11.2%) and *Cell wall/membrane/envelope biogenesis* (M, 12%). Most of the *Prevotella* genomes carry significantly (p < 0.05) higher percentage of singletons involved in *Replication, recombination and repair* (L, 12%, s.d. = 7%), and *Cell wall/ membrane/ envelope biogenesis* (M, 12%, s.d. = 7%) COG category [Additional file 8: Figure S4]. The COG category *Transcription* (K) is also quite well represented in certain UGT isolates like *Prevotella denticola CRIS 18C-A*, *Prevotella oralis ATCC 33269, Prevotella timonensis CRIS 5C-B1,* all three GIT isolates and certain oral isolates like *P. melaninogenica ATCC 25845*, *P. nigrescens, P. multiformis* etc. In our analysis we found one interesting observation that COG category *amino acid transport and metabolism (E)* is significantly overrepresented (10%, p = 0.033) in only *P. denticola F0289* out of all 28 *Prevotella* genomes.

### Comparative analysis of gene repertoire of *P. denticola CRIS 18C-A* and *P. denticola F0289*

The genetic architecture of the *Prevotella* members of the microbiome might have been influenced by the specific environment of the respective body site of the host. In an attempt to have a better insight into such niche-specific divergences, we have carried out a comparative analysis of two strains of same species *P. denticola*: *P. denticola CRIS 18C-A* and *P. denticola F0289* isolated from the different habitats - urogenital tract and oral cavity respectively.

**Figure 8:**
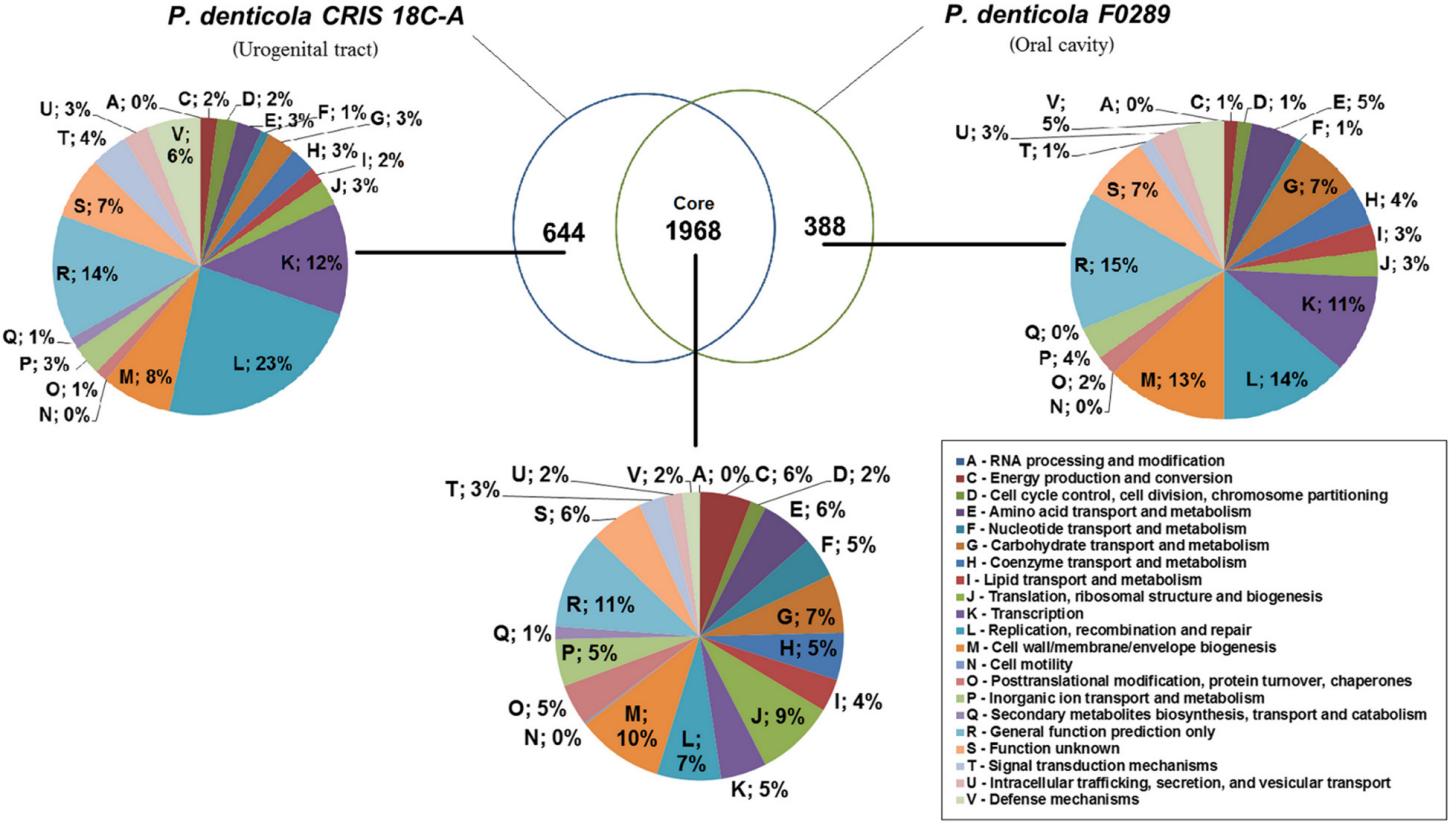
**Comparative functional analysis of gene repertoire of** ***P. denticola CRIS 18C-A*** **and** ***P. denticola F0289*** **strains.**

Both *P. denticola* strains share 1968 genes (Figure 8). 221 COG annotated genes out of 644 strain specific genes of UGT isolate *P. denticola CRIS 18C-A* are enriched in *Replication, recombination and repair* (L, 23%) and *Signal transduction mechanisms* (T, 4%) compared to 140 COG annotated genes out of 388 strain specific genes of oral isolate *P. denticola F0289.* On the other hand, the percentage occurrence of genes associated with *Carbohydrate transport and metabolism* (G) is much higher (7%) in the oral isolate, as compared to that (3%) in the UGT isolate.

All CDSs shared between two strains of *P. denticola* were subjected to the test for positive selection. Out of 1968 shared genes, only six genes were appeared to be under positive selection (Additional file 9: Table S5). The positively selected genes are under special evolutionary pressure. It is usually believed that genes undergoing positive selection in a genome might have some special functional significance.

## Discussion

Microorganisms, especially the ones adapted to specialized life-style and/or environment often exhibit niche-driven divergence or convergence in genome composition [2]. Adaptive molecular strategies of microbes implicate diverse molecular processes like horizontal acquisition of beneficial genes or genome islands, elimination of superfluous genes through reductive evolution, genetic recombination, positive selection in certain genes and so on [1-9].

One of the major objectives of the current report was to investigate whether there are any specific gene families that are exclusively present or absent from the genomes derived from a specific site of the human body. The rationale behind this objective is that such exclusively present/absent gene-families might have some roles in adaptation at their respective habitats. The current analysis of 28 *Prevotella* genomes as a case study is the first report on the habitat-specific differences in gene and COG repertoires in closely related microbiota. In the original report on reference genomes of human microbiome pan-genome analysis was carried out at species-level for 4 GIT isolates *Lactobacillus reuteri*, *Bifidobacterium longum*, *Enterococcus faecalis* and *Staphylococcus aureus*, but no attempt has been made to identify habitat-specific variations in gene-contents in these genomes [16].

This study has revealed not only the exclusive presence, but also exclusive absence of certain gene families in genomes derived from specific body sites of hosts. There are 11798, 3673, 3348 and 934 gene families present exclusively in *Prevotella* genomes derived from human oral cavity, GIT, UGT and skin, respectively. On the other hand, there are 17, 221, 115 and 645 gene families specifically absent in oral cavity, GIT, UGT and skin isolates of PDGHM. Hence, it appears that adaptive strategies of *Prevotella* might have involved “gain-of-functions” as well as “loss-of-functions” through acquisition/removal of selected genes that might have facilitated their colonization at specific body habitats. Trends in distribution of various functional COG categories differ appreciably among the habitat-specific genes. Presence of accessory as well as unique genes from the *Signal transduction mechanisms* category in relatively high frequencies among the skin and GIT isolates of *Prevotella*, or higher frequencies of genes in the category of *Defense mechanism* among singletons of the UGT isolates and some oral derivatives; clearly point towards the niche-specific modulation in genetic make-ups of microbiome components. Future investigations on metabolic/physiological implications of niche-specific COG distribution may provide clue to the host-microbiome interactions at different body sites.

The pan-genome of PDGHM has already exceeded nine times the average genome size of a typical *Prevotella* species of the dataset and still remains open. On an average, 17% of predicted protein-coding genes of any particular *Prevotella* genome represent the conserved core genes, while the remaining 83% contribute to the flexible and singletons. Such a huge repertoire of dispensable genes (i.e., accessory and unique genes) conforms well with the inter-genome variations in the number of the predicted CDSs, which varies from 1935 to 3337 (Table 1) and it suggests that each *Prevotella* genome under study might have its own genetic machinery implicated for host adaptation.

As expected, distribution of functional categories among the core genes showed the categories of typical house-keeping genes like *Translation, ribosomal structure & biogenesis*, *Nucleotide transport & metabolism*, *Energy production & conversion* etc. On the other hand, higher occurrences of genes pertaining to *Cell wall/membrane/envelope biogenesis*, *Replication, recombination & repair* and *Transcription* processes among the singletons concur well with the concept that the “variable” or “dispensable” parts of any genome usually contain non-essential and/or regulatory genes, especially the ones that would facilitate the adaptation of the respective species to any specific environment or life-style.

Presence of a huge number of singletons suggests a pivotal role of lateral gene transfer in shaping the *Prevotella* members of the human microbiome. An examination of the distribution profiles of the average GC-contents of the core, accessory and unique genes of individual organisms revealed that the core genes and most of the accessory genes have the GC-contents within a range of 35-55%, but a substantial fraction of the singletons have GC-content >50%, which complies with the notion of horizontal acquisition of these genes (data not shown).

One of the *Prevotella* members in our dataset, *P. melaninogenica ATCC 25845* is known to possess two chromosomes [35]. With a view to examine the bias, if any, in the distribution patterns of core genes, of accessory genes and especially of the singletons in these two chromosomes, we have analyzed their gene content. The primary chromosome of *P. melaninogenica ATCC 25845* contains 1318 (~57%) coding genes, out of them 314, 930 and 74 belong to core, accessory and unique genome respectively, while, the secondary chromosome contains the remaining 978 (~43%) coding genes, out of them 142, 682 and 98 belong to core, accessory and unique genome respectively. Intriguingly, the larger chromosome shares less (74) unique genes while smaller chromosome shares more (98) unique genes. Distribution of functional COG categories on primary chromosome and secondary chromosome do not differ significantly (p < 0.05) among core and accessory genes of *P. melaninogenica ATCC 25845* but in case of unique genes, COG category *carbohydrate transport and metabolism* (G, 8.1%, p = 0.0107), and *coenzyme transport and metabolism* (H, 6.75%, p = 0.0351) have significantly higher representation on secondary chromosome.

Despite substantial niche-specific renovations in the genetic makeup of the microbiota components, their taxonomic identities have not been perturbed; as suggested by clear separation of *Bacteroides* and *Prevotella* genomes in the pan-genome-based tree (Figure 3B), core genome based tree (Figure 3B) and in the clusters formed on the basis of KEGG pathways or COG repertoires (Figure 4 and Figure 5). If niche specialization would have dominated over taxonomic trends, the GIT-derived genomes from both *Prevotella* and *Bacteroides* should have co-segregated together in these cases, but this didn’t happen. These observations indicate that the human microbiota at different body habitats tend to develop their molecular adaptive strategies in such a way that the *in situ* niche-specific modulations in their gene repertoires do not have any global impact on their taxonomic characteristics.

One intriguing observation is conspicuous gene repertoire of the oral isolate *P. tannerae*. It contains a highest percentage of unique genes. Its inclusion led to a sharp decrease in the number of core genes in Additional file 3: Figure S2 and it segregated away from other *Prevotella* genomes both in the 16S ribosomal RNA-based tree (Figure 3A) and in the binary gene presence/absence matrix-based tree (Figure 3B). These observations are in good agreement with the recent reassignment of *P. tannerae* under a novel genus *Alloprevotella gen. nov.* [34]. At the time of initiation of this study, *P. tannarae* was known to be a member of the genus *Prevotella* and hence, it was included in the PGDHM dataset. Later, it was reclassified under a novel genus [34], but we have neither discarded it from the dataset, nor changed its previous name, as we intended to examine whether it emerges out as an out-group species in our analysis. In fact, spontaneous segregation of *P. tannerae* (renamed as *A. tannerae*) away from other *Prevotella* species in Figure 3B re-confirmed that the adaptive, niche-specific trends in gene acquisition and elimination in these microbiome members do not override their taxonomic trends, in general.

Clustering of genomes on the basis of distribution of KEGG pathways (Figure 4) or that of functional COG categories (Figure 5) did not exhibit any major habitat-specific segregation of the *Prevotella* genomes. Though these findings apparently do not conform to the observations on the co-segregation of certain habitat-specific genomes in pan-matrix or core genome based trees, these are consistent with the findings of The Human Microbiome Project Consortium that metagenomic carriage of metabolic pathways was stable among individuals despite variations in the community structure [36].

There were three species *P. buccae, P. denticola* and *P. melaninogenica*, each of which had two strains in the dataset. In all three trees, constructed on the basis of 16S rRNA sequences, binary gene presence/absence matrix and core genome, the strains of the same species segregated together (Figure 3A-C). This observation indicated that the intra-species variations in genome composition are smaller than inter-species variations, in general and this is true even in cases of two strains of *P. denticola* derived from different body sites – the oral cavity and UGT- of the host. Comparative analysis of the gene repertoires in two strains of *P. denticola* revealed substantial divergences in gene repertoire across two strains, especially in those belonging to the *Replication, recombination and repair*, *transcription* and *Carbohydrate transport and metabolism* categories. However, on the basis of comparative genome analysis of only two strains, it is not possible to infer whether these differences indeed represent any habitat-specific evolutionary trends, or they are mere standard deviations usually observed between any two strains of a species.

The present study points out the selective acquisition and elimination of gene families; those might have some important role in adaptation of specific *Prevotella* strains at different body habitats.

An analysis of synonymous and non-synonymous substitution rates in orthologs across two strains of *P. denticola* identified a small number of positively selected genes (Additional file 9: Table S5), but in order to arrive at any definite conclusion on importance of the process of positive selection in niche-adaptation in *Prevotella*, one would need more genome information on strains of a particular species adapted to different body habitats.

One may argue that the *Prevotella* draft genome sequences used in the study were derived from a cohort study and hence, it is not clear whether the diversity in gene content across the genomes represent a variation within (alpha-diversity) or across (beta-diversity) individuals. Furthermore, the PDGHM dataset used in the current analysis contains only 28 *Prevotella* genomes included in the catalog of initial reference genomes, released by Human microbiome consortium in 2010 [16]. With inclusion of more genomes of the genus *Prevotella* in the dataset, the number of niche-specific genes (present/absent) as well as of unique genes may alter significantly. One cannot rule out the possibility of missing some bona fide genes lying in the unfinished genomes, however small these parts may be. But, the probability that same genes would be missed exclusively from all genomes derived from a specific habitat is very low. Nevertheless, the niche-specific trends identified in the present study are quite intriguing and it underscores the need of further in-depth pursuit of such habitat-specific signatures in microbiome-derived genomes. Needless to say, characterization and interpretation of habitat-driven variations in genetic frameworks of closely related microbiota may provide a deep insight into the major trends in adaptive molecular evolution in resident microbes and help comprehending the genetic basis of competitive exclusion between microbes in response to local environmental perturbations within the host.

## Conclusions

*Prevotella* might have developed distinct genetic strategies for adaptation to different anatomical habitats within human body through selective, niche-specific modulation of gene repertoire. Each individual microbe may also develop a component of its own, distinctive genetic stratagem for host adaptation, as appeared from the huge number of singletons. Even two strains of the same species, isolated from distinct body habitats may have notable genomic divergences, as exemplified by comparative analysis of two strains of *P. denticola*. But, the niche specific genomic modulations may not override the lineage-specific divergences of the microbiome components. Open pan-genome of *Prevotella* suggests the need for sequencing more and more *Prevotella* strains, which will provide clear view of complete gene pool of *Prevotella.*

## Methods

### Sequence retrieval

All available whole genome sequences belonging to the genus of *Prevotella* (as on January, 2013) were downloaded from Data Analysis and Coordination Center (DACC) (http://www.hmpdacc.org/HMRGD/) for the National Institutes of Health (NIH) Common Fund supported Human Microbiome Project (HMP) to construct a dataset of “*Prevotella* Draft Genomes from Human Microbiome” (PDGHM) [37]. *Prevotella* genomes under this study were stated as Finished, High Quality Draft or Improved High Quality Draft in HMP project catalog. HMP sets the standards for High Quality Draft genome (N50 > 5 kb for contigs or > 20 kb for scaffolds and percentage bacterial core gene set > 90%) to ensure completeness of the genomes. N50 is a weighted median statistic such that 50% of the entire assembly is contained in contigs or scaffolds equal to or larger than this value [38,39]. We also calculated completeness of draft assembly for each *Prevotella* genome based on N50 score (Table 1) using QUAST server [40] and percentage occurrence of orthologs of bacterial core gene set (Table 1) of 200 universal core genes among bacteria [38,39]. Based on these calculations, we concluded that all draft assemblies are endowed with a very high quality (near to complete genome).

There were 28 genomes from 25 *Prevotella* species - three species, namely *P. buccae, P. denticola* and *P. melaninogenica*, had the genomes of two strains each and other 22 species have only one strain each in the dataset. Among 28 genomes of PDGHM dataset, 17 were isolated from the oral cavity, 7 from urogenital tract, 3 from gut and 1 from skin of the human body. There were total 73864 protein sequences that formed the working dataset of PDGHM.

### Protein clustering (creation of protein families)

All 73864 protein sequences of PDGHM were subjected for clustering using CD-HIT Suite using 50% identity and 50% sequence coverage as control parameters [32,41]. 24885 distinct clusters (gene families) were formed. The paralogs were removed from all clusters. The clusters were then processed using in-house perl programs to create a binary matrix (also called pan-matrix) where, rows represent clusters and columns represent respective genomes in ‘1’, ‘0’ format, i.e. ‘1’ for presence and ‘0’ for absence of the genes from respective genome, in each cluster.

### Plots for pan-genome, core genome

The number of clusters after addition of each new genome was plotted as a function of the number of genomes added sequentially in all possible permutations of the order of addition using distance guide (DG) algorithm in PanGP software [42]. The power-law regression ( *y*_*pan*_ *= A*_*pan*_*x*^*Bpan*^ *+ C*_*pan*_ ) was used to model the pan-genomes generated from all permutations, where *y*_*pan*_ is the total number of gene families in the pan-genome, *x* is the number of genomes considered, *A*_*pan*_, *B*_*pan*_ and *C*_*pan*_ are fitting parameters [33,42]. When 0 < *B*_*pan*_ < 1, the pan-genome should be considered open because it is an unrestrained function over the number of genomes. When *B*_*pan*_ < 0, the pan-genome is considered closed since it approaches a constant as more genomes are considered.

Number of core genes after addition of each new genome was plotted as a function of the number of genomes added sequentially, in similar manner as pan-genome plot. The exponential curve fit model, *y*_*core*_ *= A*_*core*_*e*^*Bcore.x*^ *+ C*_*core*_ was used to fit the core genome. Here, *y*_*core*_ denotes core genome size, *x* denotes number of genomes; *A*_*core,*_*B*_*core*_ and *C*_*core*_ are fitting parameters.

### Phylogenetic reconstructions

Three phylogenetic trees were constructed based on binary pan-matrix, core genome and 16S rRNA sequences.

The pan-matrix was used to construct Neighbor Joining Tree using PAST 3 [34], with 100 bootstrapping replications, concatenated alignments of conserved core gene sequences from all genomes were used to construct a Neighbor Joining Phylogenetic Tree. Both Core genome tree and 16S ribosomal RNA tree were constructed using MEGA6 with 1000 bootstrapping replications [43]. TreeGraph 2 was used to format trees [44].

### COG protein function analysis and KEGG pathway analysis

Protein functions were predicted by using RPSBLAST program against COG database of prokaryotic proteins and metabolic pathways were predicted by blast search against KEGG database, via WebMGA server [45].

### Codon usage distance calculations

Codon usage distances between core gene sets of all genomes were calculated using the formula:

$$ {d}_{i,j}={\displaystyle \sum_{t= all\kern.2em  coding\kern.2em  triplets}^n{\left({f}_{t,i}-{f}_{t,j}\right)}^2} $$

Here, *d*_*i,j*_ is the codon usage distance between core gene sets of *i*^th^ and *j*^th^genome, *f*_*t,i*_ and *f*_*t,j*_ are the relative codon frequencies of respective genomes [46].

### Test for positive selection between two *P. denticola* strains

We estimated values of dN (non-synonymous substitutions) and dS (synonymous substitutions), within each pair of core genes of *P.denticola CRIS 18C-A* and *P.denticola F0289* using in-house program. The pairs which show dN/dS > 1 (an excess of non-synonymous substitutions over synonymous ones) were considered to be under positive selection [47].

## Additional files

Additional file 1: Figure S1. The gene family frequency spectrum for 28 *Prevotella* genomes. Bars represent the number of orthologous gene families belonging to singletons (17166), flexible genome (7263) and core genome (456). Additional file 2: Table S1. Details of genes belonging to core, flexible and unique genome of each *Prevotella* strain. Additional file 3: Figure S2. (a) Pan and core genome plot of 28 *Prevotella* genomes. The plot shows progression of core and pan-genome after sequential addition of *Prevotella* genomes into the analysis as per their body niche. The color bars represent the number of new gene families added into the *Prevotella* pan-genome. The species names are colored according to their niches. (b) Trends of core and pan-genome curves with variation in order of genomes. Variation in shape of pan and core genome curve due to consideration of *Prevotella* genomes in different orders based on body niche, the arrow indicates the variation in pan and core genome size after addition of *P. tannerae* genome into the analysis. Letters indicate niches (g: GIT, o: ORAL Cavity, s: SKIN and u: UGT). Additional file 4: Table S2. Details of genes present in *Prevotella* strains of specific niche. Additional file 5: Table S3. Details of genes absent from *Prevotella* strains of specific niche. Additional file 6: Table S4. Details of genes exclusively absent from each *Prevotella* strain. Additional file 7: Figure S3. Heatmap of codon usage distances between core genomes of *Prevotella* strains. Additional file 8: Figure S4. Relative abundance and distribution pattern of COG categories within singletons. The colored number above pie chart represents niches of respective *Prevotella* strain (brown: GIT, green: ORAL Cavity, purple: SKIN and blue: UGT). Additional file 9: Table S5. Details of genes under positive selection.

## Abbreviations

GIT: Gastro-intestinal tract
UGT: Urogenital tract
PDGHM: *Prevotella* draft genomes from human microbiome
CDSs: Coding sequences
HMP: Human microbiome project
