# Supplementary material for: Divergences in gene repertoire among the reference Prevotella genomes derived from distinct body sites of human
Source: BMC Genomics. 2015 Mar 5;16(1):153. doi: 10.1186/s12864-015-1350-6 (PMC4359502; doi:10.1186/s12864-015-1350-6)
Supplement: Additional file 1: Figure S1. — The gene family frequency spectrum for 28 Prevotella genomes. Bars represent the number of orthologous gene families belonging to singletons (17166), flexible genome (7263) and core genome (456). [file 12864_2015_1350_MOESM1_ESM.pdf]

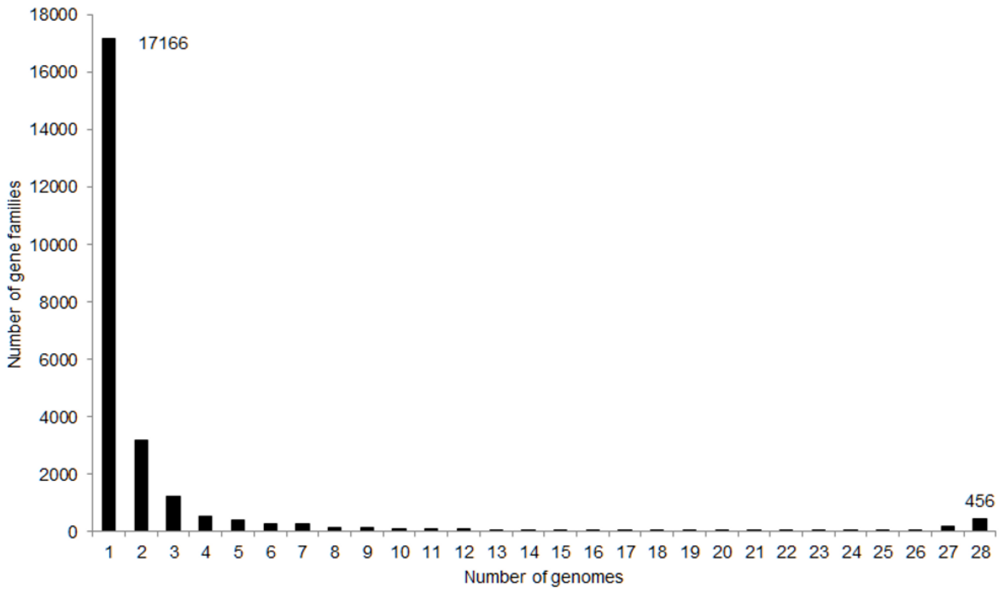

Additional File 1: Figure S1 - The gene family frequency spectrum for 28 *Prevotella* genomes. Bars represent the number of orthologous gene families belonging to singletons (17166), flexible genome (7263) and core genome (456).
