# Supplementary material for: Divergences in gene repertoire among the reference Prevotella genomes derived from distinct body sites of human
Source: BMC Genomics. 2015 Mar 5;16(1):153. doi: 10.1186/s12864-015-1350-6 (PMC4359502; doi:10.1186/s12864-015-1350-6)
Supplement: Additional file 3: Figure S2. — (a) Pan and core genome plot of 28 Prevotella genomes. The plot shows progression of core and pan-genome after sequential addition of Prevotella genomes into the analysis as per their body niche. The color bars represent the number of new gene families added into the Prevotella pan-genome. The species names are colored according to their niches. (b) Trends of core and pan-genome curves with variation in order of genomes. Variation in shape of pan and core genome curve due to consideration of Prevotella genomes in different orders based on body niche, the arrow indicates the variation in pan and core genome size after addition of P. tannerae genome into the analysis. Letters indicate niches (g: GIT, o: ORAL Cavity, s: SKIN and u: UGT). [file 12864_2015_1350_MOESM3_ESM.pdf]

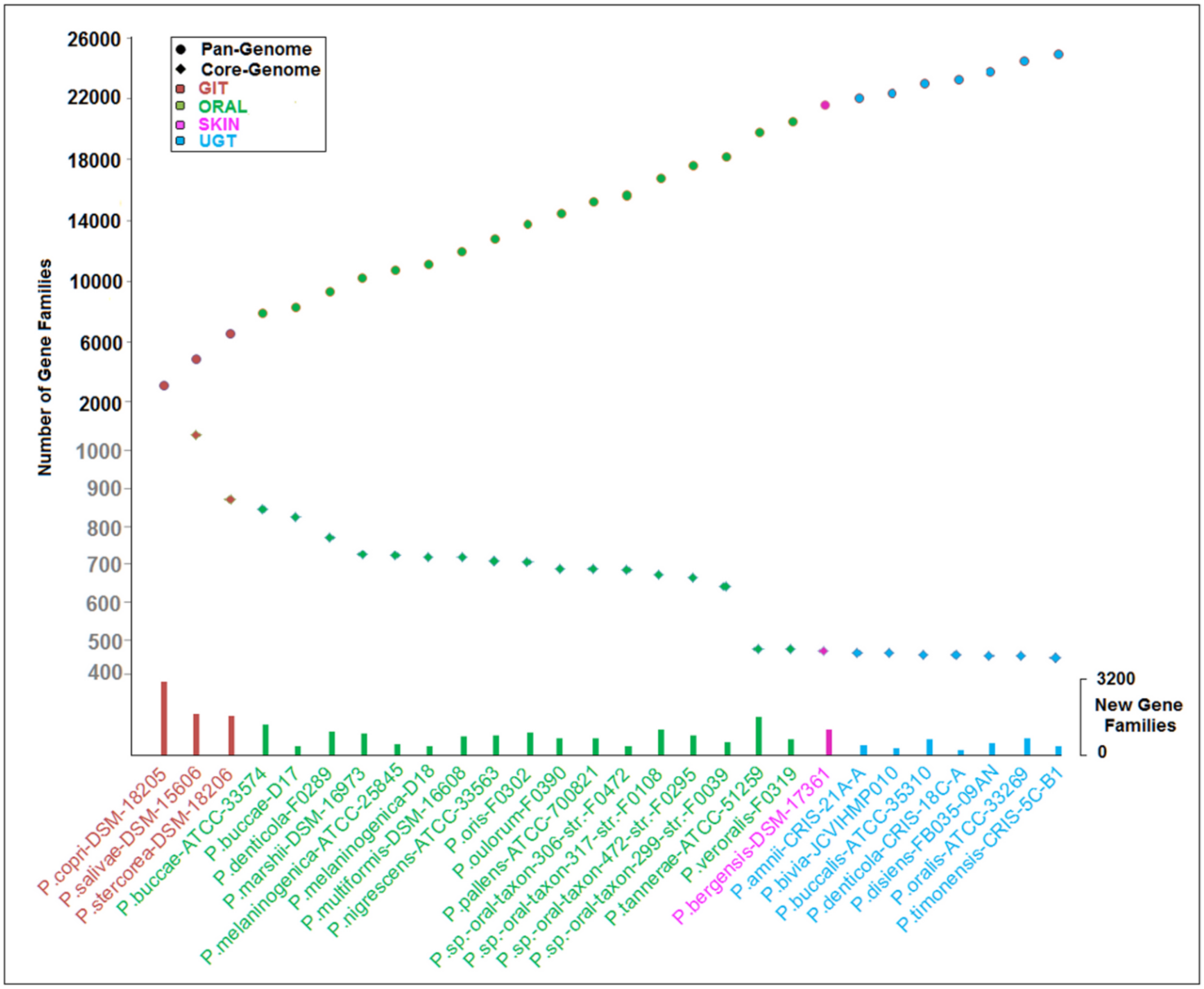

Additional File 3: Figure S2a - Pan and core genome plot of 28 *P revotella* genomes. The plot shows progression of core and pan-genome after sequential addition of *Prevotella* genomes into the analysis as per their body niche. The color bars represent the number of new gene families added into the *Prevotella* pan-genome. The species names are colored according to their niches.

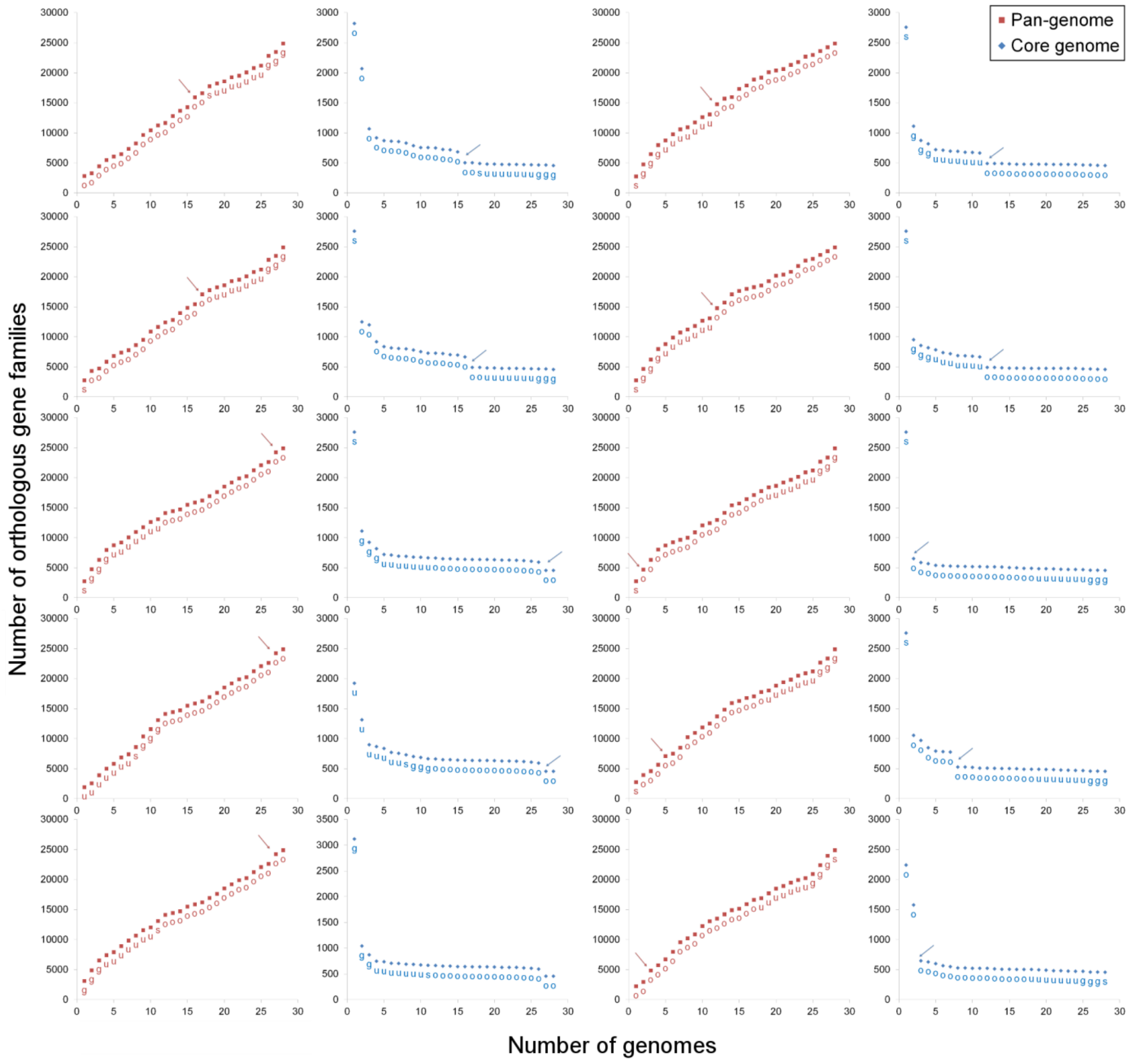

Additional File 3: Figure S2b - Trends of core and pan-genome curves with variation in order of genomes. Variation in shape of pan and core genome curve due to consideration of *Prevotella* genomes in different orders based on body niche, the arrow indicates the variation in pan and core genome size after addition of *P. tannerae* genome into the analysis. Letters indicate niches (g: GIT, o: ORAL Cavity, s: SKIN and u: UGT).
