# Supplementary material for: Divergences in gene repertoire among the reference Prevotella genomes derived from distinct body sites of human
Source: BMC Genomics. 2015 Mar 5;16(1):153. doi: 10.1186/s12864-015-1350-6 (PMC4359502; doi:10.1186/s12864-015-1350-6)
Supplement: Additional file 6: Table S4. — Details of genes exclusively absent from each Prevotella strain. [file 12864_2015_1350_MOESM6_ESM.pdf]

Additional File 6: Table S4 Details of genes exclusively absent from each *Prevotella* strain.

Niche	Organism name	Protein Id (EMBL)	Gene Description	COG Id	COG Class
**GIT**	*P.copri DSM 18205*	EFC74560.1	phosphopyruvate hydratase	COG0148	G
	P.stercorea DSM 18206	EFM00833.1	chaperone protein ClpB	COG0542	O
		EEX52238.1	arabinose 5-phosphate isomerase	COG0794	M
		EFZ37143.1	undecaprenyl-diphosphatase UppP	COG1968	V
		EFB93670.1	rubredoxin	COG1773	C
		EGC86300.1	3-deoxy-8-phosphooctulonate synthase	COG2877	M
		AEA20996.1	GDP-mannose 4,6-dehydratase	COG1089	M
		EGC86225.1	3-deoxy-D-manno-octulosonate cytidylyltransferase	COG1212	M
		EFB32658.1	GDP-L-fucose synthase	COG0451	MG
		AEA20365.1	conserved hypothetical protein	-	-
**SKIN**	*P.bergensis DSM 17361*	EHJ42052.1	pyridine nucleotide-disulfide oxidoreductase family protein	COG2509	R
		EFB92986.1	class II glutamine amidotransferase	COG0034	F
**UGT**	*P.amnii CRIS 21A-A*	EFU29670.1	large conductance mechanosensitive channel protein	COG1970	M
		EID33434.1	IgA Peptidase M64	-	-
	P.buccalis ATCC 35310	EFB33325.1	3-deoxy-manno-octulosonate-8-phosphatase	COG1778	R
		EFB93929.1	ribosomal protein L30	COG1841	J
		EFB32494.1	ribosomal protein L27	COG0211	J
	P.disiens FB035-09AN	EEX53829.1	glycine-tRNA ligase	COG0423	J
	P.oralis ATCC 33269	EFN92164.1	firmicute fructose-1,6-bisphosphatase	COG3855	G
	P.timonensis CRIS 5C-B1	EFB34908.1	2-oxoglutarate oxidoreductase, beta subunit	COG1013	C
		EFU31583.1	2-oxoglutarate ferredoxin oxidoreductase subunit gamma	COG1014	C
		EFB33144.1	branched-chain-amino-acid transaminase	COG0115	EH
		EFU31581.1	2-oxoglutarate ferredoxin oxidoreductase subunit alpha	COG0674	C
**ORAL**	*P.buccae D17*	EFC69904.1	cytochrome D ubiquinol oxidase, subunit II	COG1294	C
		EFA98590.1	NADH:ubiquinone oxidoreductase, Na(+)-translocating, A subunit	COG1726	C
		EFU30246.1	DNA polymerase III, gamma/tau subunit DnaX	COG2812	L
		ADK96346.1	adenylate kinase	COG0563	F
		EGQ17448.1	ATP-dependent protease LonB	COG0466	O
		EFB93293.1	ribosomal protein L20	COG0292	J
		EFB33331.1	GTP-binding protein LepA	COG0481	M
		EGC85472.1	phosphotransferase enzyme family	COG1660	R
		EGC86374.1	citrate transporter	COG1055	P
	P.marshii DSM 16973	EGQ11584.1	rubrerythrin	COG1592	C
	P.melaninogenica D18	EFN90746.1	CTP synthase	COG0504	F
		EFB32999.1	DNA polymerase III, alpha subunit	COG0587	L
	P.oris F0302	EFZ36112.1	excision endonuclease subunit UvrA	COG0178	L
	P.oulorum F0390	EFA97784.1	peptidase C1-like family protein	COG3579	E
		EFB34185.1	D-phosphoglycerate dehydrogenase	COG0111	HE
		EFM02180.1	conserved hypothetical protein	COG4198	S
		EFB92161.1	putative phosphoserine transaminase	COG1932	HE
		EFC74778.1	glutamate dehydrogenase	COG0334	E
	P.sp. oral taxon 317 str. F0108	EFU31314.1	3-oxoacyl-(acyl-carrier-protein) synthase II	COG0304	IQ
		EFB33724.1	maltodextrin phosphorylase	COG0058	G
	P.sp. oral taxon 472 str. F0295	EFA97313.1	ABC transporter, ATP-binding protein	COG1137	R

	EFA91775.1	carboxynorspermidine decarboxylase	COG0019	E
	EFZ38106.1	fumarate reductase subunit B	COG0479	C
	EFC74986.1	alpha-glucosidase	-	-
P.sp. oral taxon 299 str. F0039	EFA97265.1	ribosomal protein L17	COG0203	J
	AEA21872.1	S-ribosylhomocysteinase LuxS	COG1854	T
	EGQ18946.1	50S ribosomal protein L31	COG0254	J
	EFC67472.1	conserved hypothetical protein	-	-
P.tannerae ATCC 51259	EFA98549.1	1,4-dihydroxy-2-naphthoate octaprenyltransferase	COG1575	H
	EFC75848.1	3'-5' exonuclease domain protein	COG0349	J
	EFU30634.1	5'/3'-nucleotidase SurE	COG0496	R
	EGQ14933.1	50S ribosomal protein L29	-	-
	EFM02034.1	50S ribosomal protein L9	COG0359	J
	EFC76794.1	acetyltransferase	COG0456	R
	EGV30218.1	adenylate cyclase	COG2954	S
	EFV05134.1	aminodeoxychorismate lyase	COG1559	R
	EFA98345.1	ApbE family protein	COG1477	H
	EGQ23346.1	arginine repressor	COG1438	K
	EFU29992.1	ATP-dependent Clp protease ATP-binding subunit ClpC	COG0542	O
	EFU30366.1	band 7/Mec-2 family protein	COG0330	O
	EFL47097.1	biotin-requiring enzyme	COG4770	I
	EFC74124.1	carboxyl- protease	COG0793	M
	EEX52666.1	cardiolipin synthase	COG1502	I
	EGQ16917.1	cardiolipin synthetase	COG1502	I
	EFC74084.1	cell division protein FtsA	COG0849	D
	EFV05759.1	cyclic nucleotide-binding domain protein	COG0664	T
	EGQ21498.1	cytidine deaminase	COG0295	F
	EFA97739.1	deoxyribose-phosphate aldolase	COG0274	F
	EGQ17357.1	dihydrodipicolinate synthase	COG0329	EM
	EID33252.1	DNA polymerase III, delta subunit	COG0470	L
	EFA92488.1	DNA polymerase III, delta subunit	COG1466	L
	EGC20754.1	DNA primase	COG0358	L
	EFU31466.1	DNA processing protein DprA	COG0758	LU
	EFB36474.1	DNA repair protein RecN	COG0497	L
	EEX51824.1	DNA-directed DNA polymerase III beta subunit	COG0592	L
	EFA98000.1	efflux ABC transporter, permease protein	COG2177	D
	EGC86910.1	efflux transporter, RND family, MFP subunit	COG1566	V
	EGC85489.1	endonuclease/exonuclease/phosphatase family protein	COG2374	R
	EGQ18182.1	endonuclease/exonuclease/phosphatase family protein	-	-
	EFA91710.1	exodeoxyribonuclease VII, large subunit	COG1570	L
	EFV05431.1	Fe-S oxidoreductase	COG0621	J
	AEA22275.1	FHA domain protein	-	-
	EFA97916.1	gliding motility-associated protein GldE	COG1253	R
	EFC74972.1	glutamine amidotransferase subunit PdxT	COG0311	H
	EFB35041.1	glutamine cyclotransferase-related protein	COG2234	R
	EFC76889.1	glutathione peroxidase	COG0386	O
	EFB33431.1	glycosyl hydrolase, family 25	COG3757	M
	EFB32745.1	glycosyl transferase, group 1 family	COG0438	M
	EEX54059.1	group 2 glycosyl transferase	COG0463	M
	EGQ17815.1	GSCFA family protein	-	-
	EFA91757.1	HD domain protein	COG1418	R

EFB31567.1	HDIG domain protein	COG1480	R
EFC73587.1	HimA protein	COG0776	L
EFL45536.1	hydrolase, TatD family	COG0084	L
EFB33339.1	inner membrane protein OxaA	COG0706	U
EFC75811.1	iron compound ABC transporter, permease protein	COG4139	H
EFA91929.1	lipid kinase, YegS/BmrU family	COG1597	IR
EFC73130.1	lipoate-protein ligase B	COG1235	R
EFC72943.1	lipoprotein	COG1196	D
EFC75823.1	lipoprotein	COG3147	S
EFB30907.1	mandelate racemase/muconate lactonizing enzyme family protein	COG4948	MR
EFN92151.1	mannosyl-glycoprotein endo-beta-N-acetylglucosaminidase	COG1705	NU
EFC72248.1	membrane protein	COG0392	S
EFC75286.1	membrane protein	COG4591	M
EFC75604.1	membrane protein	COG0628	R
EFC76492.1	metallo-beta-lactamase family protein	COG0491	R
EFZ37246.1	MOP/MATE family multidrug-resistance efflux pump	COG0534	V
EID33320.1	MORN repeat protein	COG4642	S
EFC74704.1	MotA/TolQ/ExbB proton channel family protein	COG0811	U
EFB33091.1	multidrug resistance protein, FusA/NodT family	COG1538	MU
EFZ35886.1	Na(+)-translocating NADH-quinone reductase subunit C	COG2869	C
EEX18935.1	Na/Pi cotransporter family protein	COG1283	P
EFB31922.1	NAD dependent epimerase/reductase-related protein	COG0451	MG
EGQ12105.1	NADH dehydrogenase subunit J	COG0839	C
EGQ16369.1	naphthoate synthase	COG0447	H
EFB34324.1	octaprenyl-diphosphate synthase	COG0142	H
EFC74994.1	O-methyltransferase family protein	COG4122	R
EFB92717.1	outer membrane assembly lipoprotein YfiO	COG4105	R
EFB32498.1	outer membrane protein, OMP85 family	COG4775	M
EFB93480.1	PAP2 family protein	-	-
EFU30040.1	patatin family phospholipase	COG4667	R
EFA92212.1	penicillin-binding protein, transpeptidase domain protein	COG0768	M
EFC76200.1	peptidase, M16 family	COG0612	R
EFB93678.1	peptidase, M23 family	COG0739	M
EFC75739.1	peptidyl-prolyl cis-trans isomerase	-	-
AEA20066.1	permease, YjgP/YjgQ family	COG0795	R
EFC74938.1	phenylacetic acid degradation-related protein	COG2050	Q
EFB32504.1	polysaccharide export protein, BexD/CtrA/VexA family	COG1596	M
EFB93212.1	potassium uptake protein, TrkH family	COG0168	P
EGQ15145.1	preprotein translocase	COG1862	U
EGQ15221.1	primosome assembly protein PriA	COG1198	L
EFV04290.1	prolipoprotein diacylglyceryl transferase	COG0682	M
EFA97696.1	protein-export membrane protein SecD	COG0342	U
EFB31615.1	putative cell division protein FtsQ	-	-
AEA20944.1	putative membrane protein	-	-
EFB92356.1	putative tetraacyldisaccharide-1-P 4'-kinase	COG1663	M
EFC76991.1	putative TPR domain protein	-	-
EGQ21579.1	pyridoxal biosynthesis lyase PdxS	COG0214	H
EID33506.1	rare lipoprotein B family protein	-	-

	EFB32542.1	RecF protein	COG1195	L
	EFB93251.1	RNA methyltransferase, RsmE family	COG1385	S
	EFA96944.1	RNA polymerase sigma-54 factor	COG1508	K
	EFB92316.1	rod shape-determining protein MreC	COG1792	M
	EFU29995.1	septum formation protein Maf	COG0424	D
	ADK95980.1	Ser/Thr phosphatase family protein	COG0737	F
	EFC75794.1	shikimate dehydrogenase	COG0169	E
	EFZ38209.1	shikimate kinase	COG0703	E
	ADK95333.1	sigma-54 interaction domain protein	COG2204	T
	EFC67405.1	sigma-70, region 4 family	COG1595	K
	EEX18471.1	signal peptide peptidase SppA, 67K type	COG0616	OU
	EEX51805.1	sodium:solute symporter family protein	COG0591	ER
	EFC75605.1	thymidine kinase	COG1435	F
	AEA20380.1	translocator protein, LysE family	COG1280	E
	EGC86277.1	transport energizing protein, ExbD/TolR family	COG0848	U
	EFA92087.1	transporter, CPA2 family	COG0475	P
	EGQ17955.1	trigger factor	COG0544	O
	EFC76508.1	tRNA pseudouridine synthase A	COG0101	J
	EGQ17140.1	UDP-N-acetylglucosamine 2-epimerase	COG0381	M
	EGQ13350.1	YitT family protein	COG1284	S
	EFA93064.1	hypothetical protein	COG1253	R
	EGQ16766.1	hypothetical protein	COG4485	S
	EGV34473.1	hypothetical protein	-	-
	EGC85215.1	hypothetical protein	-	-
	EFV04101.1	hypothetical protein	-	-
	EGC85722.1	hypothetical protein	-	-
	EEX52279.1	hypothetical protein	-	-
	EGQ16284.1	hypothetical protein	-	-
	EFU31816.1	hypothetical protein	-	-
	EFV03578.1	conserved hypothetical protein	COG1738	S
	EFV05107.1	conserved hypothetical protein	COG0795	R
	EEX52500.1	conserved hypothetical protein	COG5146	H
	EEX52791.1	conserved hypothetical protein	COG1739	S
	EFB93539.1	conserved hypothetical protein	COG4980	R
	AEA21846.1	conserved hypothetical protein	COG2957	E
	EFB35318.1	conserved hypothetical protein	COG3150	R
	EFU30209.1	conserved hypothetical protein	-	-
	EFU29947.1	conserved hypothetical protein	-	-
	EFA97114.1	conserved hypothetical protein	-	-
	EFV03296.1	conserved hypothetical protein	-	-
	EFB30761.1	conserved hypothetical protein	-	-
	EFN91071.1	conserved hypothetical protein	-	-
	EEX18957.1	conserved hypothetical protein	-	-
	EFV05576.1	conserved hypothetical protein	-	-
	EFA92802.1	conserved hypothetical protein	-	-

GIT: Gastrointestinal Tract, Oral: Oral Cavity, UGT: Urogenital tract.
