# Supplementary material for: Divergences in gene repertoire among the reference Prevotella genomes derived from distinct body sites of human
Source: BMC Genomics. 2015 Mar 5;16(1):153. doi: 10.1186/s12864-015-1350-6 (PMC4359502; doi:10.1186/s12864-015-1350-6)
Supplement: Additional file 7: Figure S3. — Heatmap of codon usage distances between core genomes of Prevotella strains. [file 12864_2015_1350_MOESM7_ESM.pdf]

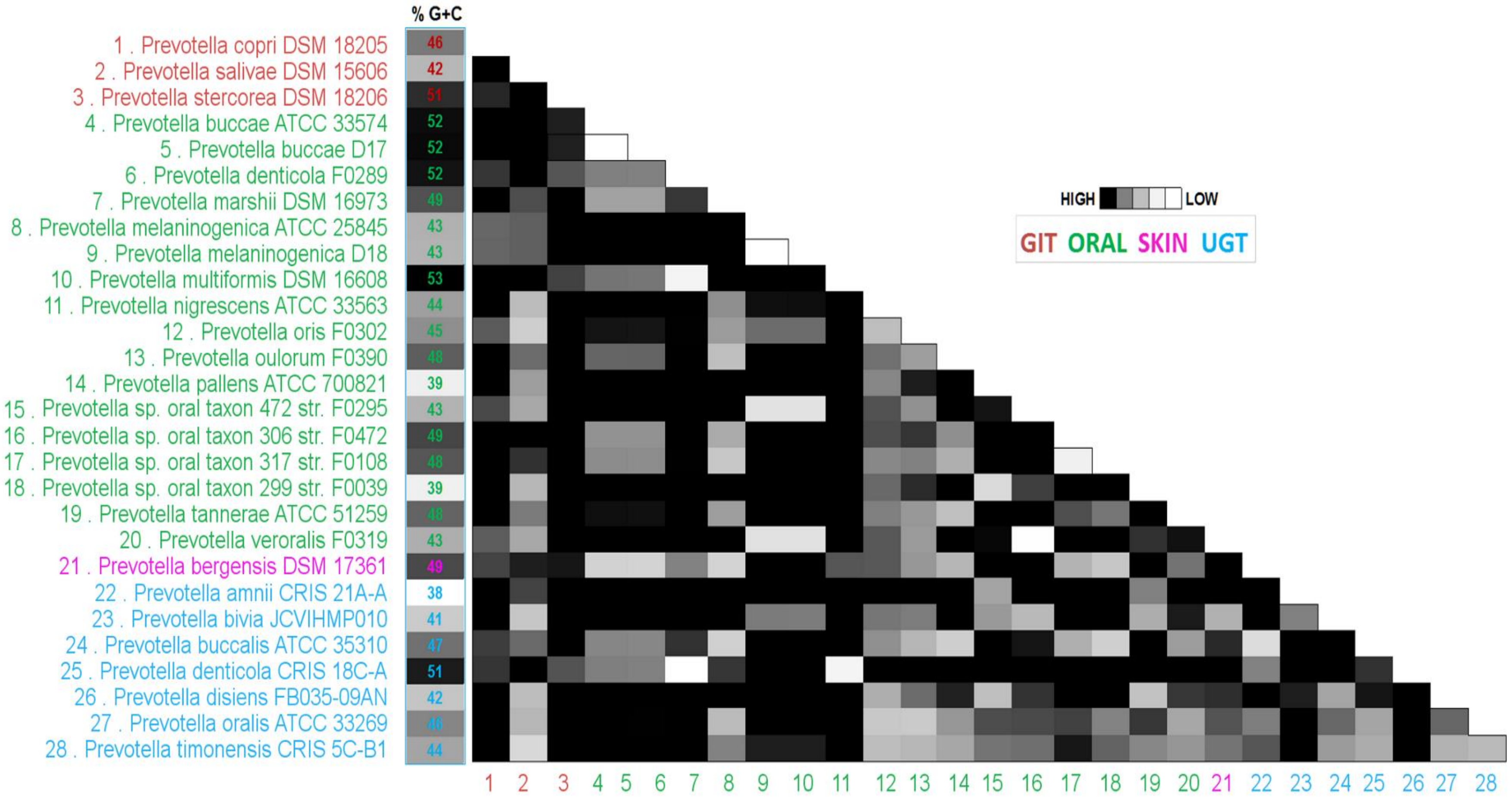

Additional File 7: Figure S3 Heatmap of codon usage distances between core genomes of *Pre votella* strains.
