# Supplementary material for: Divergences in gene repertoire among the reference Prevotella genomes derived from distinct body sites of human
Source: BMC Genomics. 2015 Mar 5;16(1):153. doi: 10.1186/s12864-015-1350-6 (PMC4359502; doi:10.1186/s12864-015-1350-6)
Supplement: Additional file 8: Figure S4. — Relative abundance and distribution pattern of COG categories within singletons. The colored number above pie chart represents niches of respective Prevotella strain (brown: GIT, green: ORAL Cavity, purple: SKIN and blue: UGT). [file 12864_2015_1350_MOESM8_ESM.pdf]

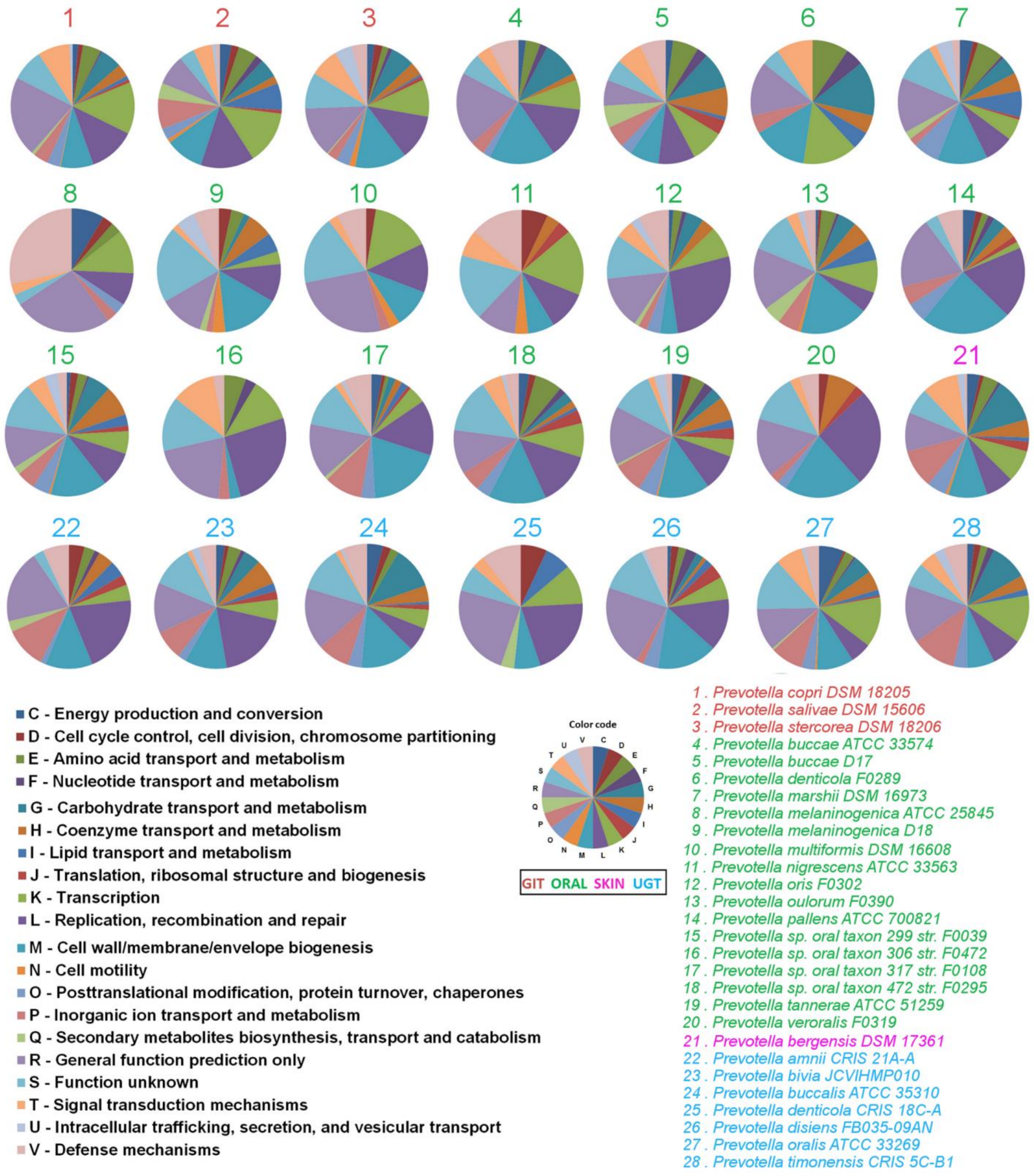

Additional File 8: Figure S4 – Relative abundance and distribution pattern of COG categories within singletons. The colored number above pie chart represents niches of respective *Prevotella* strain (brown: GIT, green: ORAL Cavity, purple: SKIN and blue: UGT).
